# Chemical Features of Polyanions Modulate Tau Aggregation and Conformational States

**DOI:** 10.1021/jacs.2c08004

**Published:** 2023-02-08

**Authors:** Kelly M. Montgomery, Emma C. Carroll, Aye C. Thwin, Athena Y. Quddus, Paige Hodges, Daniel R. Southworth, Jason E. Gestwicki

**Affiliations:** † Department of Pharmaceutical Chemistry, University of California San Francisco, San Francisco, California 94158, United States; ‡ The Institute for Neurodegenerative Diseases, University of California San Francisco, San Francisco, California 94158, United States; § Department of Biochemistry and Biophysics, University of California San Francisco, San Francisco, California 94158, United States

## Abstract

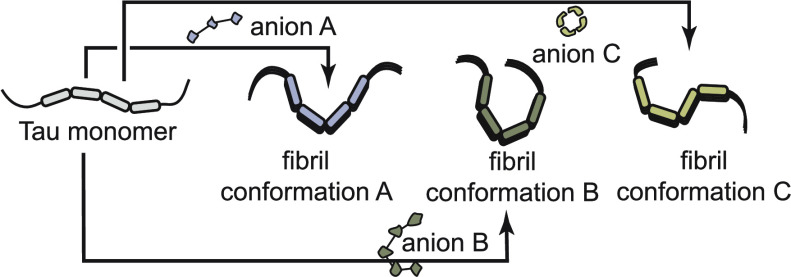

The aggregation of tau into insoluble fibrils is a defining feature of neurodegenerative tauopathies. However, tau has a positive overall charge and is highly soluble; so, polyanions, such as heparin, are typically required to promote its aggregation *in vitro*. There are dozens of polyanions in living systems, and it is not clear which ones might promote this process. Here, we systematically measure the ability of 37 diverse, anionic biomolecules to initiate tau aggregation using either wild-type (WT) tau or the disease-associated P301S mutant. We find that polyanions from many different structural classes can promote fibril formation and that P301S tau is sensitive to a greater number of polyanions (28/37) than WT tau (21/37). We also find that some polyanions preferentially reduce the lag time of the aggregation reactions, while others enhance the elongation rate, suggesting that they act on partially distinct steps. From the resulting structure–activity relationships, the valency of the polyanion seems to be an important chemical feature such that anions with low valency tend to be weaker aggregation inducers, even at the same overall charge. Finally, the identity of the polyanion influences fibril morphology based on electron microscopy and limited proteolysis. These results provide insights into the crucial role of polyanion–tau interactions in modulating tau conformational dynamics with implications for understanding the tau aggregation landscape in a complex cellular environment.

## Introduction

**Figure 1:**
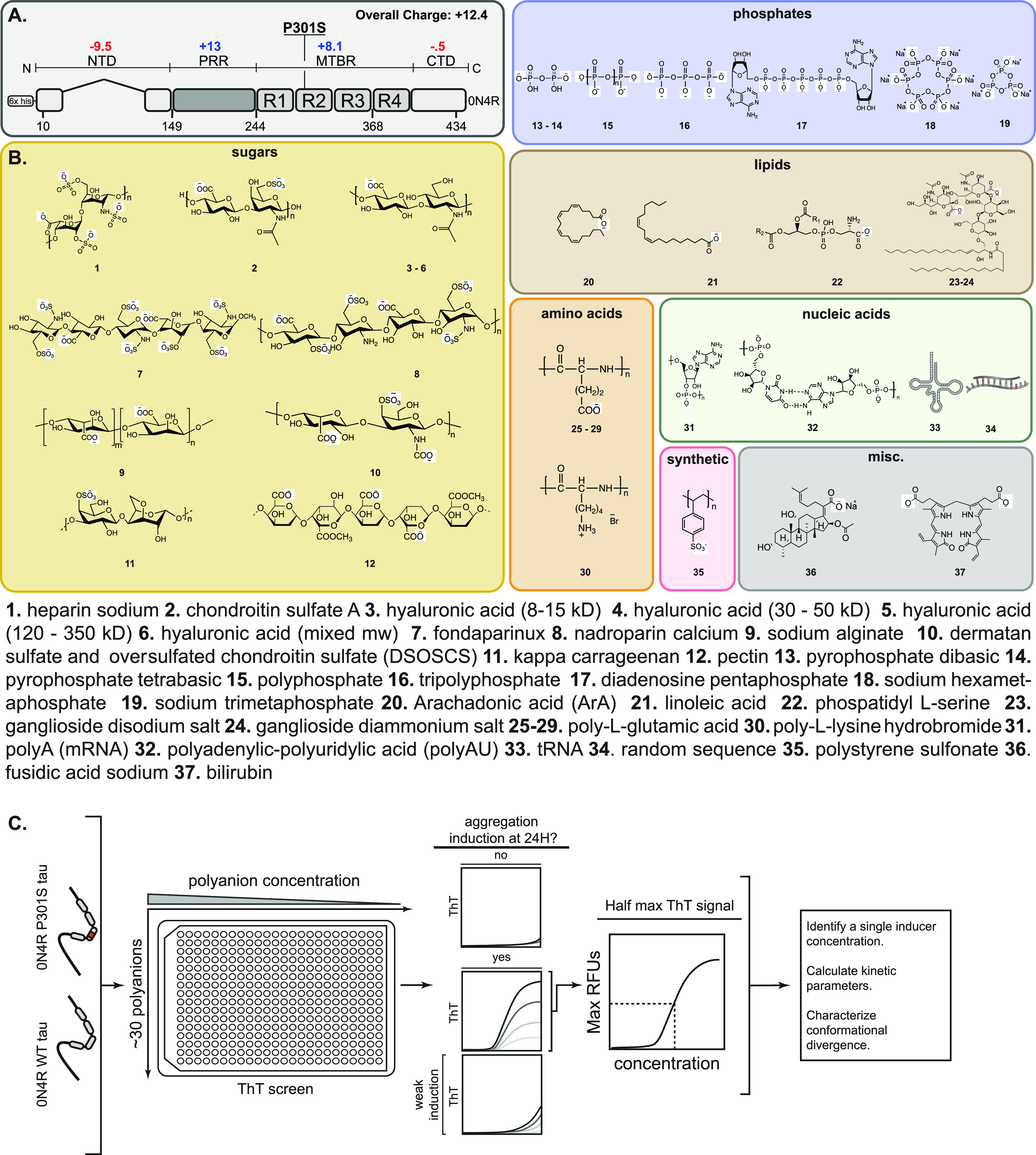
Workflow for testing the effects of a polyanion library on tau self-assembly. (A) Domain architecture of the common adult isoform of tau (0N4R). The overall charge of the domains is indicated, and the location of the his-tag and the P310S missense mutation are shown. N-terminal domain (NTD); proline-rich region (PRR); microtubule-binding repeats (MTBR); C-terminal domain (CTD). (B) Chemical structures of 37 anionic biomolecules, grouped by series. When appropriate, the minimal repeating unit is shown, and the average polymer length (*n*) is indicated in the Supplementary Tables 1 and 2. Compounds **33** and **34** are shown as cartoons because they do have repeating structures (see the Experimental Section). (C) Workflow for screening the anion library. Briefly, 0N4R tau (WT) and 0N4R P301S mutant tau (P301S) proteins were first tested against a range of concentrations of each library member in ThT assays. Anions were excluded from further analysis if they produced artifacts (Supplementary Figure 1) or if they failed to produce a ThT signal 40 RFUs above baseline fluorescence (ΔRFU ≤ 40) at 24 h. For the remaining molecules, the half-maximal effective concentration (EC_50_) was determined, and subsequent kinetic studies were performed at that anion concentration. From those studies, kinetic parameters, including lag time and elongation rate, were determined.

The class of neurodegenerative disorders known as tauopathies, including Alzheimer’s disease (AD), cortical basal degeneration (CBD), and progressive supranuclear palsy (PSP), is characterized by the accumulation of insoluble protein aggregates in the brain.^1,2^ These aggregates are primarily composed of microtubule-associated protein tau (MAPT/tau), an intrinsically disordered protein that is expressed as a series of six distinct splice isoforms.^3,4^ Tau’s isoforms are composed of a variable number of N-terminal domains (0N, 1N, or 2N), a proline-rich domain, and either three or four microtubule-binding repeats (3R or 4R; Figure 1A). The common adult isoform of tau, 0N4R, is strongly cationic at physiological pH, with an isoelectric point of ∼9.5. Accordingly, it has been known for decades that purified tau is highly soluble and not prone to spontaneously self-associate *in vitro*, even at extremes of pH and temperature.^5^ Rather, tau aggregation is typically initiated by the addition of polyanionic biomolecules, such as heparin sodium (HS), which leads to relatively rapid self-assembly.^6^ It is thought that polyanions reduce charge repulsion between cationic tau monomers, allowing the juxtaposition of aggregation motifs within the R repeats.^7,8^ These observations suggest that polyanions could also be involved in the initiation of tau aggregation *in vivo*. In support of this idea, HS is associated with tau pathology in patient brains^9^ and unresolved densities are observed in some purified, patient-derived tau fibril samples, which are hypothesized to be anions or salts.^10,11^ At present, we do not know the identity of the critical natural anion(s) or how their chemical properties might contribute to tau aggregation or fibril structures.

Structural studies have revealed that the core of tau can adopt a variety of conformations within fibrils; for instance, postmortem brain slices derived from patients with PSP and CBD contain tau fibrils with distinct folds.^12−15^ Could the identity of the anion help dictate these specific conformations? To reach the fibril state, tau is known to transition through intermediate structures, including oligomers.^16,17^ While many factors likely contribute to the eventual structural differences between fibrils,^18−20^ we hypothesize that the polyanion could help guide early stages of the self-assembly process and contribute to determining the final structure. Indeed, an increasing body of evidence suggests that polyanions have an effect on early stages of fibril nucleation *in vitro*.^21−23^ Yet, only a limited number of anions, including those in the broad categories of sugars,^8^ fatty acids,^24^ nucleic acids,^25^ and phosphates,^26,27^ have been studied for their ability to promote tau self-assembly and a systematic study, involving direct comparisons between these varied molecules under the same experimental conditions, is lacking.

Here, we collected 37 chemically and structurally diverse anions and tested them side-by-side in a thioflavin T (ThT) assay to identify those that mediate tau’s aggregation. We find that a surprisingly large number of anionic biomolecules, including sugars, polypeptides, nucleic acids, amino acids, and lipids, promote this process. Valency appears to be an important feature of these molecules because only polyanions of sufficient repeat length were able to promote fibril formation. We also find that a disease-associated mutant, P301S tau, is sensitive to a larger number of anions (28/37) than the wild type (21/37), which could be one reason why it is linked to severe diseases. A subset of the inert anions were found to inhibit tau aggregation in the presence of heparin. To explore the potential impact of polyanions on the conformation of tau fibrils, we selected some of the most potent inducers and explored the resulting fibrils by limited proteolysis, sedimentation, and transmission electron microscopy (TEM). Remarkably, we find that the identity of the polyanion has a dramatic effect on tau fibril conformation. Together, these results expand our knowledge of the role of polyanions in tau aggregation *in vitro.* Based on these findings, we speculate that the chemical composition of specific anions is one important factor in shaping induction potential and fibril conformation.

## Experimental Section

### Recombinant Protein Expression and Purification

The gene encoding human 0N4R tau was cloned into a pET-28a vector and transfected into *Escherichia coli* BL21(DE3) competent cells. Starter cultures were grown in Luria broth (LB) containing 50 μg/mL kanamycin overnight at 37 °C with constant shaking at 200 rpm. Then, 20 mL of the starter culture was used to inoculate 1 L of Terrific broth (TB), containing 50 μg/mL kanamycin. Cells were grown at 37 °C, with constant shaking, until an OD_600_ between 0.6 and 0.8 was reached. At this point, the incubation temperature was set to 30 °C, and NaCl (500 mM) and betaine (10 mM) were included in the growth medium. After 30 min, expression was induced with 200 μM IPTG for 3.5 h at 30 °C.

To purify tau, cells were pelleted and resuspended in a lysis buffer containing 20 mM MES (pH 6.8), 1 mM EGTA, 0.2 mM MgCl_2_, 5 mM DTT, and 1× cOmplete protease inhibitor cocktail (Roche). Cells were lysed by sonication and then boiled for 20 min. The lysate was clarified by centrifugation for 30 min at 30,000*g*. The clarified supernatant was dialyzed overnight into His binding buffer (1× PBS, 20 mM imidazole, 200 mM NaCl, and 5 mM β-mercaptoethanol (BME)) and purified by affinity purification.

His-tagged tau was bound to Ni-NTA resin for 1 h at 4 °C with constant mixing. The resin was washed using 500 mL of His binding buffer (1× PBS, 20 mM imidazole, 200 mM NaCl, and 5 mM BME), wash buffer 1 (1× PBS, 10 mM imidazole, 300 mM NaCl, and 5 mM BME), and wash buffer 2 (1× dPBS, 15 mM imidazole, 100 mM NaCl, and 5 mM BME), and then eluted using 30 mL of elution buffer (1× PBS, 300 mM imidazole, 200 mM NaCl, and 5 mM BME). Tau was further purified using reverse-phase HPLC, as described previously.^14^ The His tag was not removed as it was found to not interfere with the aggregation reactions.^28,29^ The protein was then lyophilized and resuspended in tau buffer (1× dPBS, 2 mM MgCl_2_, and 1 mM DTT). Protein concentration was determined using the bichinchronic acid (BCA) method. The purity was >95%, as judged by Coomassie gels (Supplementary Figure S11).

### Compound Preparation

All compounds including anions and polyanions were sourced from commercial vendors and used without further purification. See Supplementary Tables 1 and 2 for details of the catalog numbers. Each polyanion was freshly prepared in assay buffer (1× Dulbecco’s PBS, pH 7.4, 2 mM MgCl_2_, 1 mM DTT) and sterilized with a 0.2-micron filter before each experiment. Lipid inducers (arachidonic acid, linoleic acid, and phosphatidyl-l-serine) were handled similarly, except that they contained a final concentration of 5% ethanol to maintain solubility.

### Tau Aggregation and Kinetic Screening

The ThT-based aggregation screen was performed in a miniaturized, 384-well plate format.^24^ The microplates (Corning 4511) were pre-rinsed with 20 μL of 0.01% Triton-X to minimize interactions with the sides of the plate. In each well, tau (10 μM), thioflavin T (10 μM), polyanion (see Supplementary Tables 1 and 2 for concentrations) and assay buffer (Dulbecco’s PBS, pH 7.4, 2 mM MgCl_2_, 1 mM DTT) were added to each well to a total volume of 20 μL. The aggregation reaction was carried out at 37 °C with continuous shaking and monitored via fluorescence (excitation = 444 nm, emission = 485 nm, cutoff = 480 nm) in a Spectramax M5 microplate reader (Molecular Devices). Readings were taken every 5 min for at least 24 h. Each experiment was performed in triplicate wells. All components of the aggregation reaction were freshly prepared each day.

### Data Processing

For data processing, the ThT signals produced by three replicates of the tau-only controls (no inducer) were averaged and this background was subtracted from corresponding samples. These values were typically less than 5 to 10% of the overall signal. For inducers with ThT background greater than 10%, mainly chondroitin sulfate A, inducer + ThT background was further subtracted. To identify the dose of inducer for subsequent kinetic analyses, we first determined the inducer concentration required to produce a half-maximal ThT fluorescence signal by fitting the fluorescence curves to a sigmoid in GraphPad PRISM. Using the half-maximal concentration, we analyzed the kinetic parameters of tau aggregation using the Grace plotting program (http://plasma-gate.weizmann.ac.il/Grace/) and fitting the aggregation curves to the Gompertz function: , as previously described.^24^ In that equation, the lag time is defined by the inflection point, the inverse of the apparent elongation rate constant (t_i_-b). In addition, *A* represents the maximum ThT signal, and the apparent elongation rate constant is 1/*b*. For error analysis, we considered the kinetic measurements from individual experiments and used them to calculate the standard error of the mean (SEM), without considering additional error introduced by goodness of fit.

### Fibril Preparation for Proteolysis

The aggregation reaction was performed in 1.5 mL Eppendorf tubes for 48 h with constant agitation at 1200 rpm. The reactions included tau (10 μM) and inducers (see Supplementary Tables 1 and 2 for concentrations) in assay buffer (Dulbecco’s PBS, pH 7.4, 2 mM MgCl_2_, 1 mM DTT) with a total volume of 300 μL. After 48 h, the reactions were subjected to ultracentrifugation (Beckman Optima Max-XP Tabletop Ultracentrifuge) using a TLA-55 Fixed-Angle Rotor at 103,000 rcf to remove monomeric tau and excess inducer. Pelleted tau fibrils were then resuspended in 1/3–1/4 of the reaction volume, and the concentration was determined by Coomassie band intensity measured against a standard and quantified in ImageLab (BioRad).

### Limited Proteolysis

Soluble tau and freshly prepared fibrils were digested with Promega sequencing grade trypsin at a protein:protease ratio 500:1 for 60 min at 37 °C. The reaction was performed in 40 mM HEPES and 40 mM NaCl (pH 8.0). Reactions were quenched with 3× SDS loading buffer + 1 mM PMSF and immediately heat-denatured at 95 °C for 5 min. The proteolysis reactions were separated using a 4–20% polyacrylamide Tris-glycine SDS-PAGE denaturing gel (Invitrogen). Gels were transferred to nitrocellulose using a TurboBlot (BioRad) and analyzed using antibodies corresponding to several tau epitopes (anti-tau 1, 5, 13, and 4R). Mouse anti-tau 1 and 5 (Thermo) and rabbit anti-4R tau (Abcam) were prepared 1:1000 in Intercept T20 (TBS) Antibody Diluent (LiCor), and anti-tau 13 (Abcam) was prepared 1:5000. All secondary antibodies were prepared 1:10000 in 1:1 TBST and T20 (TBS) Antibody Diluent (LiCor).

### Transmission Electron Microscopy

0N4R tau^WT^ fibrils were freshly prepared as described above in the kinetic screening assay for 36 h without ThT present. The corresponding samples were pooled and subsequently immobilized on 600-mesh carbon-coated copper grids (SPI). The samples were incubated for 30 s on a glow-discharged grid, and then, the solution was removed by filter paper. Three washing steps with double distilled H_2_O were followed by three staining steps with 0.75% (w/v) uranyl formate (Electron Microscopy Sciences). The samples were imaged using a FEI Tecnai 10 operated at 100 keV. Micrograph images were recorded using a 4k × 4k CCD camera (Gatan). The fibril dimensions were measured using ImageJ software.

### Fibril Sedimentation

Tau fibrils were prepared as described in the kinetic screening assay, excluding ThT. Samples were subsequently pooled and centrifuged at 13,000 rcf for 30 min at RT. Supernatants were removed, and the insoluble material was washed with 100 μL of assay buffer (1× dPBS, 2 mM MgCl_2_, 1 mM DTT) followed by an additional 30 minute centrifugation at 13,000 rpm. Following the wash, the buffer was removed, and fresh assay buffer was added to each sample. Samples were sonicated for 10 min, mixed with 3× SDS loading buffer, and boiled for 5 min prior to analysis on a 2–20% denaturing gel. Gels were stained using Coomassie brilliant blue and imaged using a ChemiDoc Imaging system.

## Results and Discussion

### Creation of an Anion Library

In choosing anions for a chemical library, we sought to incorporate benchmark compounds, such as heparin sodium (HS), as well as molecules from a variety of structural classes that had never been previously tested for their effects on tau aggregation. Accordingly, we purchased anionic sugars (**1**–**12**), polyphosphates (**13**–**19**), short chain fatty acids (**20**–**24**), polypeptides (**25**–**30**), and oligonucleotides (**31**–**34**; Figure 1B), as well as anions from more chemically diverse classes, such as antibiotics (**36**), biladienes (**37**), and synthetic polymers (*i.e.*, polystyrene sulfonate, **35**). Only a subset of these compounds (**1**, **6**, **13**, **15**, and **31**) had, to our knowledge, been previously tested for their effects on tau aggregation. While the major goal of this panel was to sample different scaffolds, some of the library members varied in their chemical properties. For example, fondaparinux (**7**), polyphosphate (**15**), and polyadenylic-polyuridylic acid (poly-AU; **32**) have varying charge density: −10 per monomer for compound **7**, −1 per monomer for **13**, and −2 for **25** (Figure 1B). Thus, we also hoped that screening this collection might also begin to reveal chemical features important for tau aggregation.

Previous work had shown that anions employ at least two mechanisms to promote tau self-assembly. For example, HS (**1**) is an example of the polymer class of inducers, and it is decorated with sulfates from a repeating disaccharide unit (Figure 1B). HS and related compounds are thought to mitigate the unfavorable, long-range electrostatic interactions in tau, favoring hydrophobic collapse of the core.^5,30−32^ However, another mechanism is linked to monovalent anions, such as arachidonic acid (**20**), which are thought to function by first forming micelles on which tau assembles.^33−35^ In our anion library, examples of both categories are included, and we anticipated that polymers, such as sugars, nucleotides, and phosphates, might function similar to HS, while the lipids and other nonpolymers might potentially function as micelles.

### Screens Identify the Subset of Anions That Promote Tau Aggregation

To evaluate the anion library, we measured tau aggregation using a 384-well plate-based ThT platform (Figure 1C).^24^ Briefly, our goal was to first screen each anion at a range of concentrations to reveal which ones could support tau aggregation; then, we would focus on the most potent inducers to perform more detailed kinetic and structural studies. In these experiments, we employed two purified, recombinant human proteins: 0N4R tau (WT; Supplementary Table 1) and a 0N4R P301S mutant (P301S; Supplementary Table 2). P301S is a genetic mutation associated with FTD-linked Parkinsonism-17 (FTDP-17) that produces severe frontal temporal atrophy.^36^ It is known that P301S is more aggregation prone than WT in the presence of HS,^37,38^ so we reasoned that it might be more sensitive to weaker inducers.

In the initial screens, we tested each member of the anion library at a range of concentrations. These ranges (see Supplementary Tables 1 and 2) were either selected from the literature (for known inducers) or chosen empirically (for those that had not been studied previously). These experiments were performed in triplicate using the ThT signals measured every 5 min for a minimum of 24 h with shaking at 37 °C (see the Experimental Section). At the same time, we performed experiments in the absence of tau protein to reveal any anions that might produce artifacts. Indeed, this control was important because we found that gangliosides (**23** and **24**), and several nucleic acids, including tRNA (**33**), polyA (**31**), and polyAU (3**2**), produced ThT signals in the absence of protein (Supplementary Figure S1), and accordingly, they were excluded from further analysis (Supplementary Tables 1 and 2). Chondroitin sulfate A (CS; **2**) produced a relatively modest tau-independent signal that could be subtracted from the experimental samples (Supplementary Figure S1), so this inducer was carried forward into the next experiments. For the remaining anions, we placed them into three categories based on the maximum ThT signal that they produced. Those considered to be “inactive” failed to reach saturation and yielded 40 or less RFUs (ΔRFU ≤ 40) above baseline at 24 h (Supplementary Tables 1 and 2; Supplementary Figure S2). Inducers were determined to be “moderately active” if they yielded a ΔRFU > 40 but did not reach a maximum ThT signal at the highest tested concentration of the anion (Supplementary Figure S3). Finally, inducers were “active” if they produced ΔRFU > 40 with a full sigmoidal curve (Supplementary Figures S4 and S5). For these anions, we determined their relative potency by fitting the sigmoid of the dose response and determining the half-maximal value (EC_50_). Importantly, we noted that a subset of “active” anions produced a hook-like effect in their dose response; they initially promote ThT signals at lower concentrations and then become inhibitory with increased dosing (Supplementary Figure S6). For example, polystyrene sulfonate (**35**) becomes inhibitory at concentrations above 250 μg/mL. For this subset of molecules, we estimated EC_50_ using the upper inflection point as the top concentration.

**Figure 2:**
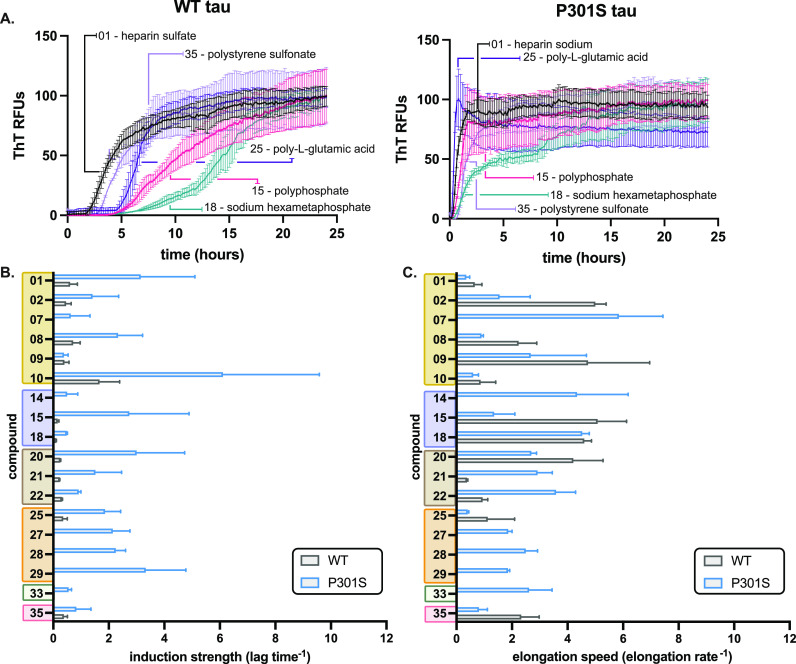
Anions have differential effects on lag time and elongation rate. (A) Representative ThT assay results, comparing heparin sodium (**1**), polyphosphate (**15**), sodium hexametaphosphate (**18**), poly-l-glutamic acid (**25**), and polystyrene sulfonate (**35**) on recombinant 0N4R WT tau (left) and 0N4R tau P301S (right) aggregation kinetics. The anions were used at their half-maximum concentration (see Supplementary Tables 1 and 2) and tau proteins at 10 μM. Results are the average of at least three independent experiments performed in triplicate, and the error bars represent SEM. For each result, the signal from control experiments using no tau was subtracted. (B) Anions have differential effects on lag time. Values are plotted as reciprocal (lag^–1^), termed the induction strength. Inactive inducers and those with weak signals were omitted from the analysis (see text). Results are the average of at least three independent experiments performed in triplicate, and the error bars represent SEM. (C) From the same aggregation reactions, the elongation rate was calculated and plotted as the reciprocal (elongation rate^–1^), termed the elongation speed. Results are the average of at least three independent experiments performed in triplicate, and the error bars represent SEM.

One of the striking results from this screen is that tau aggregation is achieved with a large number of diverse anions. More specifically, for WT tau, we find that (11/37) molecules are strongly active and (10/37) are modestly active (Supplementary Table 1). Together, these active molecules represent six out of the seven structural categories (Figure 2), suggesting that many classes of anions, with a variety of backbones, can support tau self-assembly. However, these molecules were not all equally potent. Of the molecules tested, sugars (**1**, **2**, and **8**–**10**) generally have the lowest EC_50_ values (*e.g.*, active at the lowest concentrations; Supplementary Tables 1 and 2), while phosphates (**15**–**18**) tend to be the least potent (*e.g.*, require the highest concentrations). It is important to note that these comparisons are imperfect because the natural polymers used here are heterogeneous in length and valency, so it is difficult to compare their molar concentrations. Finally, we were surprised to find that several of the anions are “inert” (unable to produce ThT signals), including hyaluronic acids (**3**–**6**), kappa carrageenan (**11**), pectin (**12**), dibasic pyrophosphate (**13**), trimetaphosphate (**19**), fusidic acid (**36**), and bilirubin (**37**) (Supplementary Tables 1 and 2). This finding suggests that charge alone is not sufficient to drive the amyloid process. We wondered whether a subset of these “inert” molecules might serve as inhibitors of other anions. To test this idea, we co-induced WT tau with heparin and a subset of the “inert” molecules. We found that some compounds, such as hyaluronic acid (**6**) and kappa carrageenan (**11**) did not interfere with HS-mediated tau aggregation (Supplementary Figure S2C). Others, such as fusidic acid (**36**) and pectin (**12**) partially blocked heparin’s activity (Supplementary Figure S2C). We reason that these inhibitory anions might partially limit heparin and other active anions from binding to tau.

In addition to having different potency values, we also find that anions produced different levels of maximal ThT signals (Supplementary Table 1 and 2). Maximum ThT fluorescence is likely a product of the number of fibrils, the number of ThT binding sites in those structures, and the chemical features of the binding sites (*e.g.*, hydrophobicity).^39^ Thus, these results begin to suggest that the conformation(s) of the fibrils formed by the different anions might be distinct.

### Kinetic Studies Reveal the Differential Effects of Anions on Lag Time and/or Elongation Rate

In aggregation reactions, the lag time is used to estimate the time required for an inducer to initiate formation of oligomers, while the elongation rate is representative of multiple steps, including monomer addition and fibril fragmentation.^40^ Thus, we reasoned that comparing these values for reactions initiated by different anions might provide further insights into the steps that are affected. In these experiments, we used each anion at or near its EC_50_ concentration, using triplicate wells and repeating the studies three times with independent tau protein samples (*n* = 9, three biological replicates). This type of comparison is important because it normalizes anion potency and allows direct comparisons between them. From the resulting data, the lag time and elongation rate were determined with the Gompertz function using the Grace plotting program (see the Experimental Section). To facilitate comparisons between anions, we plotted the reciprocal of the lag time (lag time^–1^) to calculate an “induction strength”, where higher values are indicative of faster aggregation (Figure 2). Although there is considerable variability within classes, we find that sugars generally initiate fibril formation the fastest, with lag times around 2 to 2.5 h for WT tau (Figure 2B; Supplementary Table 1). In contrast, the polyphosphates promote aggregation with considerably longer lag times (∼6 to 9 h). Next, we similarly plotted the reciprocal of the elongation rate for each reaction (Figure 2C), where high values are indicative of faster progression of fibril assembly. Again, there are differences between and within chemical classes, but sugars tend to produce the fastest elongation speeds. With both sets of values in-hand, we could then identify anions that might preferentially impact lag time or elongation rate. Indeed, we noted that HS (**1**) tended to produce dramatic effects on lag time, with comparatively little effect on elongation rate, as previously reported.^7^ However, other sugars, such as **2** and **8**, had relatively strong effects on both steps. Other compounds, such as **7** and **9**, had a disproportionate impact on elongation rate. Thus, the identity of an anion seems to determine which steps in the aggregation process are most impacted.

### Mutant P301S Is Sensitive to a Wider Range of Anions

Although we have, to this point, focused on the results obtained with WT tau, these experiments were also performed, in parallel, using mutant P301S tau. A comparison between the results obtained with these two proteins suggested that, as expected, most anions that induce weak or moderate ThT signals for WT tau give relatively stronger signals using the P301S construct, with shorter lag times (Supplementary Table 2). Indeed, for a few anions, such as **14** and **27**–**29**, aggregation was only detected with P301S. We also noted that even the cation, poly-l-lysine (**30**), which was originally selected as a negative control, was able to weakly stimulate ThT signals for P301S. Crowding agents are known to accelerate aggregation,^41^ so it is possible that poly-l-lysine might operate by this mechanism. Another unexpected result was that a small number of anions, such as **2**, **9**, **20**, and **35**, produced relatively faster elongation rates for WT vs P301S (Figure 2). Regardless, in most cases, we found that P301S was more sensitive than WT for nearly all of the anions. These findings support a model in which the strong link between this mutation and FTD could be, in part, a product of its sensitivity to a wider range of naturally occurring anions.

### Anions Require a Combination of Both Charge Density and Valency to Support Tau Aggregation

**Figure 3:**
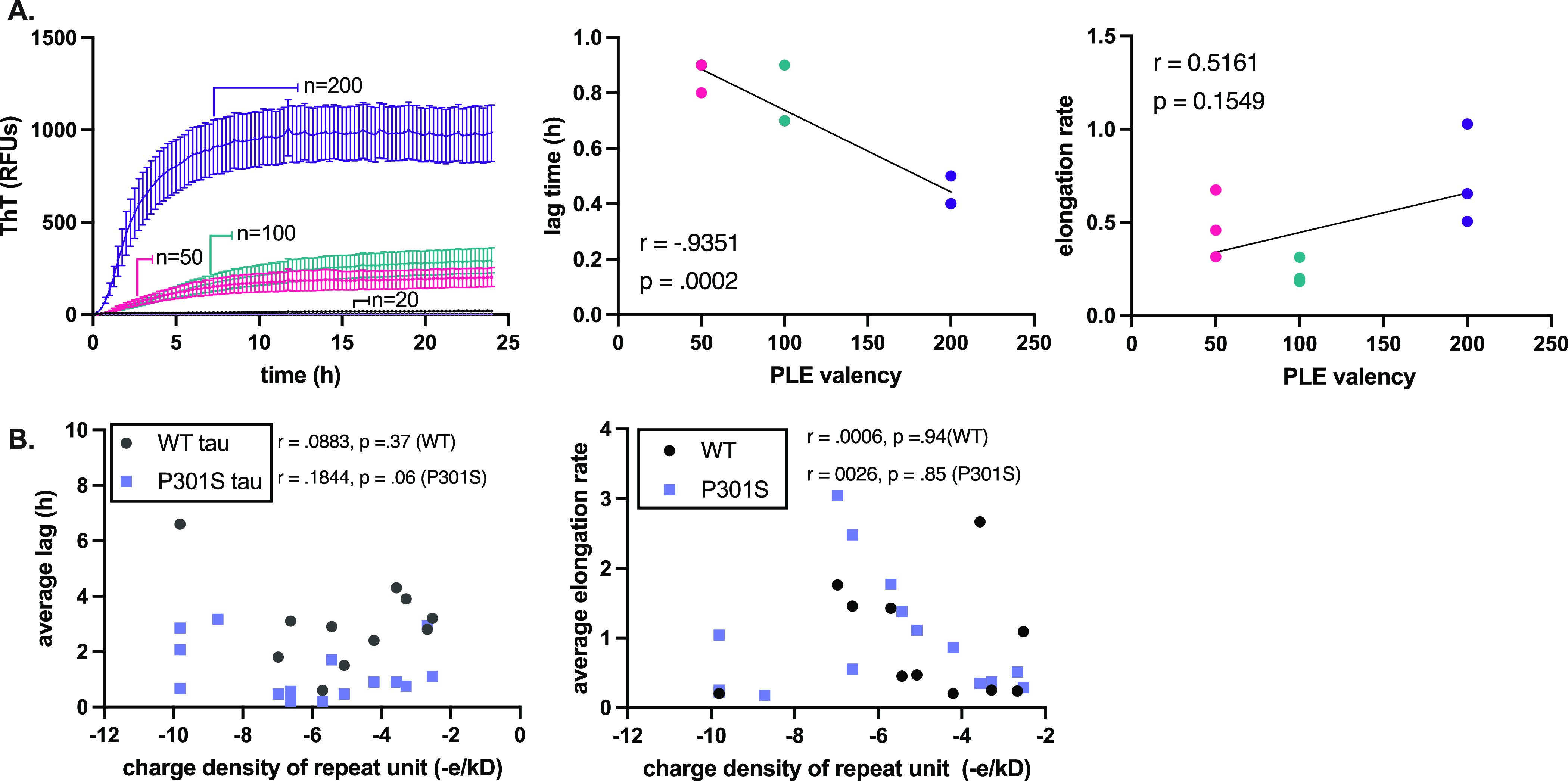
Polyanion valency is an important parameter in dictating tau fibril formation. (A) 2N solutions of poly-l-glutamic acid (PLE) of discrete molecular weights (*n* = 20, 50, 100, or 200) were used to induce WT and P301S tau (10 μM) for 24 h at 37 °C with constant shaking (left). From the resulting curves, lag time and elongation rate were extracted (right). A Pearson *t*-test was performed to measure the correlation between valency and kinetic parameters. Results are the average of at least three experiments performed in triplicate, and the error bars represent SEM (*n* = 9). Results are shown for P301S because it gives a more robust signal compared to WT tau. (B) A Pearson *t*-test was performed to determine the correlation between charge density (−e/kD) on the lag time (left) and elongation rate constants (right).

In these screens, we found that molecules from a surprising number of chemical classes could support tau assembly. Therefore, it seems likely that the individual details of the scaffold backbone (*e.g.*, sugar, peptide, *etc*.) might be relatively less important than their shared physical features, such as charge and valency. Indeed, careful studies using purified heparins and polyphosphates have pointed to a key role for valency in tau aggregation.^26^ To test this idea in more detail, we obtained additional anions, including dimeric ones, within the phosphate and amino acid chemical series and tested them in ThT assays. Within these series of chemically defined molecules, we compared their activity on a per monomer, molarity basis. The results supported the idea that anion valency seems to be an essential feature of inducers. For example, synthetic poly-l-glutamic acids (**26**–**29**) decrease lag times with increasing valency (Figure 3A; *p* value: 0.0002) but do not have a specific effect on elongation rate constants (Supplementary Figure S9; *p* value: 0.1549). Similar relationships were observed with phosphates of varying length (Supplementary Figure S9). We next wondered whether other chemical features, such as charge density, might similarly correlate with effects on lag time or elongation rate. However, plots of charge density (−e/kD or −e/Å) showed no correlation (Figure 3B; Supplementary Figure S9), suggesting that this feature is not critical. As previously suggested,^7^ we speculate that multivalent anions might bridge multiple tau monomers, increasing their local concentration and ultimately enhancing their self-assembly. However, valency is clearly not the only important feature because highly valent compounds with sparse charge, such as hyaluronic acids (**3**–**6**), are ineffective inducers, suggesting that an optimal balance of charge density and polymer valency may be important.

### Identity of the Polyanion Modulates Protease Sensitivity and Fibril Conformation

**Figure 4:**
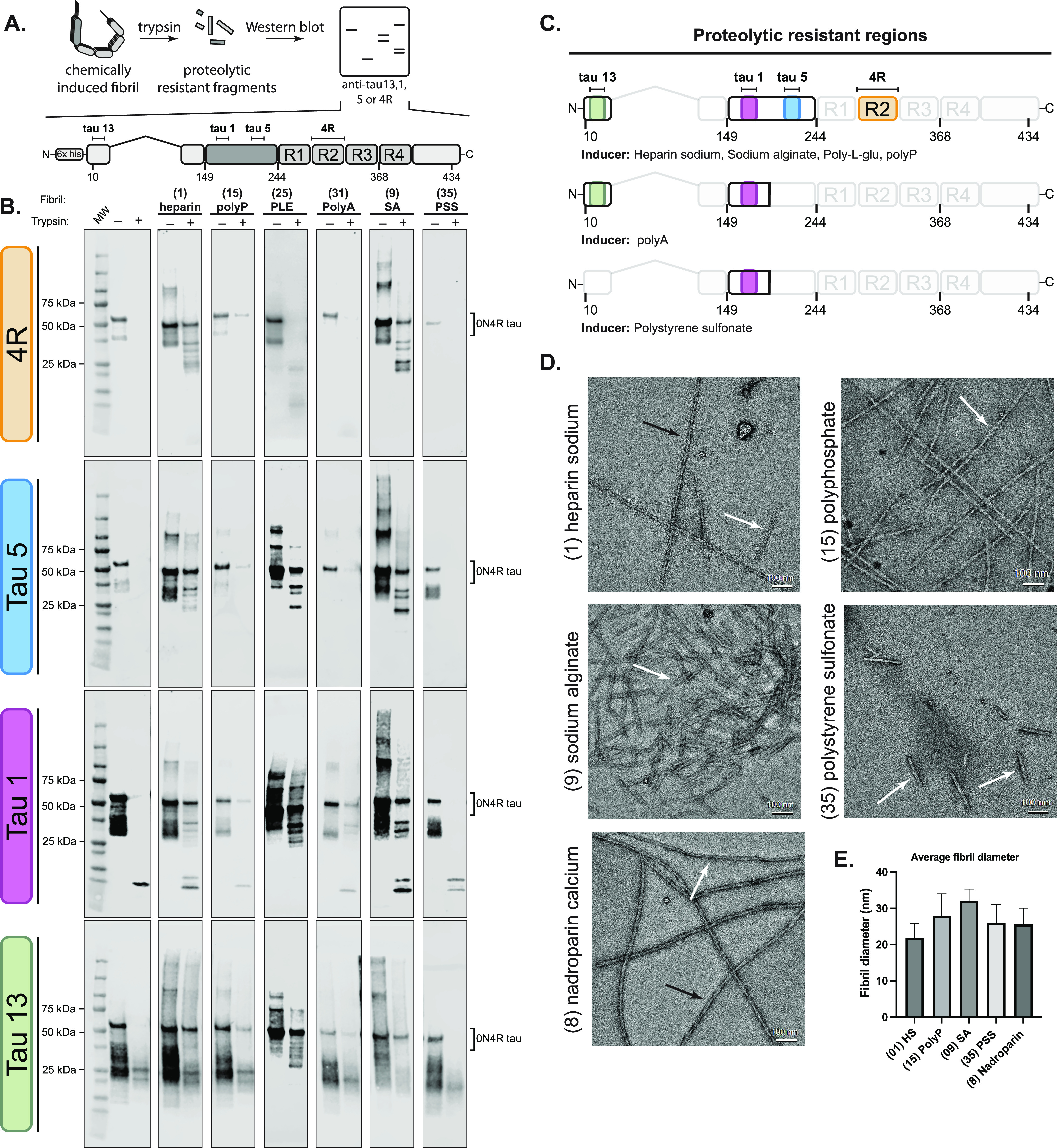
Identity of the polyanion impacts the tau fibril structure. (A) General workflow for tau fibril proteolysis. Fibrils were prepared using the corresponding inducer at its analysis concentration (see Supplementary Table 1), purified by ultracentrifugation, and subsequently proteolyzed using trypsin. Proteolysis products were separated by SDS-PAGE and probed using anti-tau antibodies (anti-tau 13, 1, 5, and 4R) (top). The domain architecture of 0N4R tau, showing the location of the epitopes for anti-tau antibodies (bottom). (B) Tau fibrils are generally resistant to proteolysis, but the digestion patterns depend on the identity of the inducer. The protease-resistant fragmentation of 0N4R WT tau filaments differentially induced using heparin (**1**), polyphosphate (**15**), poly-l-glutamic acid (**25**), polyA (**31**), sodium alginate (**9**), or polystyrene sulfonate (**35**). (C) Summary of the proteolytic resistant regions of each fibril sample. (D) Representative electron micrographs of negatively stained fibrils from recombinant 0N4R tau assembled in vitro at the end point of each reaction. Scale bar: 100 nm. Black arrows indicate twisted filaments, and white arrows indicate straight fibers. (E) Quantification of the average diameter of recombinant fibrils (*n* = 30).

In addition to their effects on assembly kinetics, we hypothesized that the identity of the polyanion might impact the structure of the fibrils. Here, we define structural conformation based on differences in susceptibility to limited proteolysis and appearance of the fibrils by negative stain transmission electron microscopy (TEM). The advantage of limited proteolysis is that it reveals potential differences in the “fuzzy coat” of tau fibrils (*e.g.*, regions outside of the well-folded core), a region that makes up the bulk of tau fibrils and has been shown to adopt heterogeneous conformers.^14,42−45^ Accordingly, WT tau fibrils formed from some of the most potent inducers, HS (**1**), polyphosphate (**15**), poly-l-glutamic acid (**25**), polyA (**31**), sodium alginate (**9**), and polystyrene sulfonate (**35**), were incubated for 60 min with the protease trypsin at a protein:protease ratio of 500:1 and then analyzed by SDS-PAGE. To distinguish fibril regions resistant to digestion, we probed these samples using several anti-tau antibodies (tau 1, tau 5, 4R, and tau 13), which have epitopes that span the domains of tau (Figure 4A).

In the limited proteolysis experiments, we observed rapid degradation of soluble tau, which is consistent with its intrinsic disorder and many trypsin cleavage sites (Figure 4b; Supplementary Figure S7). For the fibrils, we generally observed resistance to proteolysis and, more importantly, we observed differential proteolytic banding between samples. Conformational divergence is perhaps most apparent when probing the proline-rich region (PRR). For example, using the anti-tau 5 antibody (residues 210–230), three bands ranging from 25 to 37 kDa are apparent for heparin (**1**)-, poly-L-glutamic acid (**25**)-, and sodium alginate (**9**)-induced samples, whereas a single faint band persists for polyP (**15**), and no protease-resistant banding is observed in this same region using polyA (**31**) or polystyrene sulfonate (**35**). Probing further upstream in the PRR, using the anti-tau 1 antibody (residues 192–204), the initial similarities observed between **1** and **9** and **25** diverge. Specifically, heparin (**1**) and sodium alginate (**9**) maintain a similar proteolysis profile with two bands between 30 and 37 kDa, in addition to the emergence of a ∼15 kDa band. For poly-l-glutamic acid (**25**), however, the fibrils remain resistant to degradation in this region, with two intense bands appearing around 25 to 30 kDa. Interestingly, the 15 kDa fragment also appears in samples induced with polystyrene sulfonate (**35**) and this is the only region where this sample displayed protease resistance. Probing the N-terminal domain (NTD; residues 20–35) reveals that the fibrils formed using poly-l-glutamic acid (**25**) and heparin (**1**) were most resistant to digestion, whereas polyP (**15**), polyA (**31**), and sodium alginate (**9**) conferred moderate resistance, and PSS (**35**) are susceptible. This result suggests that the fibrils formed in the presence of compounds **1**, **9**, **15**, **25**, and **31** have NTDs that are oriented in a manner that completely or partially shields them from proteolysis (Figure 4C). Other anions, such as polyA (**31**) and PSS (**35**), might create fibrils or protofibrils that are considerably less stable. Together, findings using these three antibodies (tau 5, tau 1, and tau 13) support a conclusion in which inducers impact the conformation(s) of the fuzzy coat.

The R2 repeat region of tau is particularly important because previous work has shown that it is included in a subset of patient-derived core structures but excluded from others.^11,12^ Interestingly, we observe that the R2 repeat is included in the protected core of fibrils formed in the presence of heparin (**1**), which is consistent with heparin-induced tau structures,^46^ sodium alginate (**9**), poly-L-glutamic acid (**25**), and polyphosphate (**15**), but that it is excluded from those formed using polyA (**31**) or polystyrene sulfonate (**35**). Thus, it seems likely that only a subset of the inducers promote the formation of R2-containing fold. Indeed, our results with polyA (**31**) are consistent with cryo-EM evidence, which has shown that the R2 region is excluded from the cores of fibrils formed using RNA.^29^ The biological and/or structural significance of R2 positioning is not yet known, but it is interesting that the identity of the polyanion can cause dramatic changes to R2 protease sensitivity.

To confirm that the partial proteolysis samples were enriched for fibrils, we also performed sedimentation-based SDS-PAGE analysis on the soluble (S) and insoluble pellet (P) components after centrifugation. For both WT and P301S tau (Supplementary Figure S8), we confirmed that anions that are strongly active in ThT (**1**, **8**, **10**, and **15**) also shift tau into the pellet, with nearly full conversion to insoluble material. Likewise, anions determined to be weakly active (**17**) or inert (**3**–**6**) yielded incomplete conversion (Supplementary Figure S8). Thus, these sedimentation studies both corroborate the ThT assays and provide further evidence of differences in the potency of anions.

Finally, we used negative stain TEM to examine and compare the supramolecular architecture of tau fibrils. These experiments also served the added goal of independently confirming whether the measured ThT signals were due to the formation of amyloid fibrils. AD-associated tau fibrils tend to have a twisted or straight conformation by TEM, with relatively long fibrils that have an average diameter around 30 to 15 nm. Therefore, we wanted to study the fibrils formed by our anions and compare them to this benchmark. A full set of representative TEM images are shown in Supplementary Figure S10. Focusing just on those that were studied by limited proteolysis, we found that samples prepared using heparin (**1**), fondaparinux (**7**), polyphosphate (**15**) sodium hexametaphosphate (**18**), and nadroparin calcium (**8**) had characteristic amyloid morphologies (Figure 4D). Those formed from **8**, **7**, and **20** were particularly interesting as they yielded twisted and straight fibrils with an average diameter of ∼25 to 30 nm. The results with HS (**1**) are consistent with previous findings^46^ and include a mixture of twisted and straight fibrils. In contrast, filaments generated using sodium alginate (**9**) and polystyrene sulfonate (**35**) are relatively monomorphic and distinct. Fibrils formed using sodium alginate (**9**) were particularly striking in their unusual and consistent morphology; moreover, they tended to produce slightly thicker fibers (33 nm, *n* = 30) compared to the other inducers (Figure 4E). In the broader TEM screen, we also observed oligomeric (*i.e.*, spherical) and amorphous structures in samples formed from polyAU (**31**) and hyaluronic acid (**6**). These anions produced poor ThT signals, so their lack of ThT reactivity correlates well with the imaging. Together, these results support our hypothesis that polyanion identity strongly impacts the conformation of tau fibrils, such that the protein can be directed into strikingly different shapes by the chemical properties of the inducer.

## Conclusions

The role(s) of anions in tau self-assembly have remained elusive. Many pioneering manuscripts have shown that individual polyanions promote this process,^22,25,27,35,47^ with the most attention placed on heparins and polyphosphates. Here, we expand the landscape of tested anions, with a special focus on those naturally occurring ones that tau might encounter in the brain. We also tested them side-by-side to avoid interpretations that could arise due to differences in experimental conditions (*e.g.*, buffer and tau concentration). From these screens, we found that tau aggregation is enhanced by a wide variety of anions. Indeed, the most pervasive theme is that anions with dramatically different scaffolds (*e.g.*, sugar, polyphosphate, *etc.*) are capable of supporting tau self-assembly. This finding suggests that degenerate physical features, such as valency and charge, are more important than the specifics of the scaffold from which the anions are displayed (*e.g*, polymers and micelles). For example, the sparsely charged hyaluronic acids (**3**–**6**; 1 charge per repeat) and the low valency compounds (**14**, **29**, and **30**) were relatively poor at promoting fibril formation, but highly charged and multivalent molecules from many chemical series (**1**, **2**, **7**, **15**, **20**, **21**, and **35**) can induce this process. Together, these results are broadly consistent with a model in which polyanion-assisted aggregation proceeds via minimizing the electrostatic repulsion between positively charged tau monomers, allowing subsequent inter- or intramolecular scaffolding of monomers. Yet, the identity of the scaffold must also be important at some level because different anions produce distinct tau fibril morphologies. For scaffolds that have sufficient valency, it seems likely that more subtle features, such as flexibility or charge density, might then favor differential effects on lag time, elongation rate, and, ultimately, the structure of the resulting fibrils. For example, heparin sulfate (**1**) and sodium alginate (**9**) are both repeating di-saccharides, yet they produce dramatically different tau fibrils, as judged by either proteolysis or EM (see Figure 5). We speculate that the molecular details of the tau-anion contacts might be an important step in “folding” and fibril formation.

In the intact brain, soluble tau likely encounters many diverse and abundant biological molecules that bear a negative charge, including many of the proteins, lipids, nucleic acids, and metabolites tested here. Thus, it is interesting to consider the implications of the results in that context. For example, we find that the identity of the anion can have a strong impact on the structure of tau fibrils; thus, differences in the anion composition of specific brain regions could help dictate what types of fibrils are supported. In addition, it is possible that mixtures of anions, as likely encountered in complex cellular environments, could produce new structures or structures of mixed conformation. This is an important consideration because structural studies have shown that filaments formed *in vitro* using HS as an inducer do not have the same structure(s) as those isolated from patient brains.^46^ It is possible that different anions or combinations of anions might better replicate the patient-derived structures *in vitro*. Finally, we found that several polyanions, such as hyaluronic acids, are not capable of supporting aggregation at all. Similarly, low valency anions, such as triphosphate (**16**), were also remarkably poor at promoting tau aggregation and others, such as fusidic acid (**36**), are even inhibitors. This is an important observation because these anions might serve as competitors *in vivo*, binding to cationic sites on tau and buffering the protein’s interactions with other aggregation-promoting polyanions. If this supposition is true, then the relative concentrations of both active and inert and inhibitory anions might combine to dictate whether tau forms fibrils.

It is important to note that recombinant tau produced from *E. coli* was used in these studies, so the proteins are devoid of post-translational modifications (PTMs). *In vivo*, the overall charge of tau will be tuned by multiple PTMs, including phosphorylation, acetylation, and ubiquitination.^48,49^ These modifications can either add a negative charge (phosphate) or neutralize a positive one (acetylation), thus altering the isoelectric point and, in turn, adjusting tau’s sensitivity to polyanions.^50^ Indeed, recombinant tau fibrils induced using HS (**1**) are significantly different than those formed using phosphorylated tau.^51^ Future studies will be needed to deconvolute the impact of the vast number of possible PTM combinations. Likewise, mutations in tau might also impact its interactions with anions by altering the overall structural landscape of the protein. Indeed, we found that P301S tau was sensitive to a wider range of anions than WT, which might partly underlie this mutation’s important role in FTD. There are hundreds of additional mutations linked to tauopathy,^52^ which might also vary in their response to polyanions.

More broadly, electrostatic forces are essential in mediating protein–protein interactions (PPIs),^53,54^ not just those found in tau. Here, we screened a library of naturally occurring and synthetic polyanions and found striking differences in how they promote tau self-assembly. It seems likely that a similar, screening-based approach could be adopted to probe the potential roles of polyanions on other PPIs. While crowding agents, such as polyethylene glycol, are sometimes explored,^55^ systematic studies of polyanion libraries are less common.

## Supporting Information Available

Tables of anions and their properties; raw thioflavin T plots; testing of anions as inhibitors of tau aggregation; examples of anions that produce a “hook effect”; raw Western blots for the limited proteolysis experiments; results of sedimentation assays; and representative TEM images (PDF)
