# Supplementary material for: Chemical Features of Polyanions Modulate Tau Aggregation and Conformational States
Source: J Am Chem Soc. 2023 Feb 8;145(7):3926–36. doi: 10.1021/jacs.2c08004 (PMC9951223; doi:10.1021/jacs.2c08004)
Supplement: Supplementary file 1 — ja2c08004_si_001.pdf [file ja2c08004_si_001.pdf]

Supporting Information

The Chemical Features of Polyanions Modulate Tau Aggregation and Conformational States

Kelly M. Montgomery[1,2], Emma C. Carroll[2], Aye C. Thwin[2], Athena Y. Quddus[2], Paige Hodges[1,2], Daniel R. Southworth[2,3] and Jason E. Gestwicki[1,2,*]

[1]Department of Pharmaceutical Chemistry, University of California San Francisco, San Francisco, CA 94158.
[2]The Institute for Neurodegenerative Diseases, University of California San Francisco, San Francisco, CA 94158.
[3]Department of Biochemistry and Biophysics, University of California San Francisco, San Francisco, CA 94158.

Supplementary Table 1. Characteristics and results from the anion library screening against WT tau.

Supplementary Table 2. Characteristics and results from the anion library screening against P301S tau.

Supplementary Fig. S1. Identification of anions that produce Thioflavin T (ThT) artifacts in the absence of tau fibrils.

Supplementary Fig. S2. Raw curves supporting the activity of anions in the ThT assays.

Supplementary Fig. S3 A subset of anions weakly induce tau aggregation.

Supplementary Fig. S4. Raw ThT results for the anions determined to be “active” against WT tau.

Supplementary Fig. S5. Raw ThT results for the anions determined to be “active” against P301S tau.

Supplementary Fig. S6 Several anions accelerate aggregation at low concentrations, but then inhibit it at higher concentrations (producing a “hook effect”).

Supplementary Fig. S7. Full Western blots for the images shown in the partial proteolysis studies (see Figure 4).

Supplementary Fig. S8. Sedimentation assays reveal the extent of tau fibrilization.

Supplementary Fig. S9. Polyanion valency is an important parameter in dictating tau fibril formation.

Supplementary Fig. S10. Raw TEM images, supporting and extending the results from the ThT and sedimentation assays.

Supplementary Fig. S11. Purity of expressed tau proteins by Coomassie gels.

number	compound	CAS number	MW of repeat unit (Da)	MW range (Da)	charge per repeat unit	charge density (-e/kD)	charge density (-e/Å)	polymer classification	charged groups	dosing (ug/mL)	[EC50] (ug/mL)	[analysis] (ug/mL)	avg lag (hours)	avg elongation rate constant	endogenous in human systems?
1	heparin sodium salt	9041-08-1	573.40	17000 - 19000	-4	-6.98	-0.27	sugar	3 sulfates / 1 carboxyl	352 - 5.5	45.3	88	1.8	1.76	yes
2	chondroitin sulfate A ⌿Ψ	39455-18-0	475.38	6000 - 33000	-2	-4.21	-0.15	sugar	1 carboxyl / 1 sulfate	250 - 31.25	57.0	125	2.4	0.20	yes
3	hyaluronic acid (8-15 kD)	9067-32-7	402.31	8000 - 15000	-1	-2.49	-0.07	sugar	1 carboxyl	500 - 62.5	—	500	—	—	yes
4	hyaluronic acid (30-50 kD)	9067-32-7	402.31	30000 - 50000	-1	-2.49	-0.07	sugar	1 carboxyl	500 - 62.5	—	500	—	—	yes
5	hyaluronic acid (120-350 kD)	9067-32-7	402.31	120000 - 350000	-1	-2.49	-0.07	sugar	1 carboxyl	500 - 62.5	—	500	—	—	yes
6	hyaluronic acid (mixed mw)	9067-32-7	402.31	not availiable	-1	-2.49	-0.07	sugar	1 carboxyl	500 - 62.5	—	500	—	—	yes
7	fondaparinux sodium ┼	114870-03-0	1728.04	1728.04	-10	-5.79	-0.34	sugar	8 sulfates / 2 carboxyl	1000 - 31.25	—	500	—	—	no
8	nadroparin calcium Ψ	37270-89-6	591.45	—	-6	-5.07	-0.21	sugar	4 sulfates/ 2 carboxyl	1000 - 31.25	54.8	125	1.5	0.47	no
9	sodium alginate ┼ Ψ	9005-38-3	749.0	120000-190000	-2	-2.67	-0.12	sugar	2 carboxyls	1000 - 31.25	—	125	2.8	0.24	no
10	dermatan sulfate and oversulfated chondroitin sulfate	EPY0001321	475.38<br>696.52	not availiable	-2<br>-5	-4.21<br>-7.18	-0.15<br>-0.38	sugar	1 carboxyl / 1 sulfate<br>1 carboxyl / 4 sulfates	1000 - 61.25	88.4	125	0.6	1.43	yes
11	kappa carrageenan	11114-20-8	401.32	200000 - 800000	-1	-2.49	-0.08	sugar	1 sulfate	200 - 12.5	—	500	—	—	no
12	pectin	9000-69-5	353.3	50000 - 180000	-1	-2.83	-0.07	sugar	1 carboxyl	500 - 62.5	—	500	—	—	no
13	sodium pyrophosphate dibasic	7758-16-9	101.96	221.94	-1	-9.81	-0.31	phosphate	2 phosphates	4000 - 62.5	—	2000	—	—	yes
14	sodium pyrophosphate tetrabasic	7722-88-5	101.96	265.90	-1	-9.81	-0.31	phosphate	2 phosphates	4000 - 62.5	—	2000	—	—	yes
15	polyphosphate	10361-03-2	101.96	not availiable	-1	-9.81	-0.31	phosphate	1 phosphate	1000 - 125	167.0	250	6.6	0.20	yes
16	tripolyphosphate ┼	7758-29-4	101.96	367.85	-1	-9.81	-0.31	phosphate	3 phosphates	4000 - 500	—	2000	—	—	yes
17	Diadenosine pentaphosphate ┼	75522-97-3	101.96	916.37	-1	-9.81	-0.31	phosphate	5 phosphates	500 - 31.25	—	500	—	—	yes
18	sodium hexametaphosphate ┼	68915-31-1	101.96	611.77	-1	-9.81	-0.31	phosphate	6 phosphates	1000 - 15.6	—	500	9.2	0.22	no
19	sodium trimetaphosphate	7785-84-4	101.96	305.89	-1	-9.81	-0.31	phosphate	3 phosphates	4000 - 62.5	—	4000.0	—	—	no
20	Arachadonic acid	506-32-1	304.47	—	-1	-3.28	-0.12	lipid	1 carboxyl	30.5 - 1.9	—	31	3.9	0.25	yes
21	linoleic acid	60-33-3	280.45	—	-1	-3.57	-0.12	lipid	1 carboxyl	28.04 - 1.75	—	28	4.3	2.67	yes
22	phosphatidyl-l-serine	51446-62-9	792.07	—	-2	-2.53	-0.33	lipid	1 phospate / 1 carboxyl	18.5 - 1.16	—	79	3.2	1.09	yes
23	Ganglioside disodium salt ⌿	37758-47-7	1470.80	—	-2	-1.36	-0.20	lipid	2 carboxyls	1000 - 31.25	—	100	—	—	yes
24	Ganglioside diammonium salt ⌿	62010-37-1	1602.90	—	-2	-1.25	-0.20	lipid	2 carboxyls	1000 - 31.25	—	100	—	—	yes
25	poly-L-glutamic acid Ψ	26247-79-0	151.10	50000-100000	-1	-6.62	-0.33	amino acid	1 carboxyl	500 - 15.6	37.0	62.5	3.1	1.46	yes
26	poly-L-glutamic acid (n = 20)	26247-79-0	151.10	3000.0	-1	-6.62	-0.33	amino acid	1 carboxyl	300 - 4.6	—	—	—	—	yes
27	poly-L-glutamic acid (n = 50)	26247-79-0	151.10	7500.0	-1	-6.62	-0.33	amino acid	1carboxyl	301 - 4.6	—	—	—	—	yes
28	poly-L-glutamic acid (n = 100)	26247-79-0	151.10	15000.0	-1	-6.62	-0.33	amino acid	1 carboxyl	302 - 4.6	—	—	—	—	yes
29	poly-L-glutamic acid (n = 200)	26247-79-0	151.10	30000.0	-1	-6.62	-0.33	amino acid	1 carboxyl	303 - 4.6	—	—	—	—	yes
30	poly-L-lysine	25104-18-1	151.10	150000-30000	1	6.62	0.33	amino acid	1 amine	500 - 31.25	—	—	—	—	yes
31	polyA (mRNA) ⌿	26763-19-9	267.24	not availiable	-1	-3.74	-0.11	nucleic acid	1 phosphate	500 - 62.5	—	500	—	—	yes
32	Polyadenylic-polyuridylic acid (polyAU) ⌿	24936-38-7	671.40	not availiable	-2	-2.98	-0.06	nucleic acid	2 phosphates	500 - 62.5	—	500.0	—	—	yes
33	tRNA (yeast) ⌿	9014-25-9	—	25000.0	—	—	—	nucleic acid	—	500 - 15.6	—	500.0	—	—	yes
34	random nuc sequence	N8080127	—	—	—	—	—	nucleic acid	—	100 - 3.125	—	500.0	—	—	yes
35	polystyrene sulfonate Ψ	PSS4K	184.2	4800.0	-1	-5.43	-0.32	synthetic	1 sulfate	500 - 31.25	—	62.5	2.9	0.45	no
36	Fusidic acid sodium salt ⌿	751-94-0	538.7	—	-1	-1.86	-0.13	misc	1 carboxyl	1000 - 31.25	—	500	—	—	no
37	bilirubin	635-65-4	584.7	—	-2	-3.42	-0.10	misc	2 carboxyls	500 - 62.5	—	500	—	—	yes

Key

(dark yellow)	active
┼ (light yellow)	weak activator
(grey)	Inert
Ψ	hook effect
⌿	ThT reactivity

number	compound	CAS number	MW of repeat unit (Da)	MW range (Da)	charge per repeat unit	charge density (-e/kD)	charge density (-e/Å)	polymer classification	charged groups	dosing (ug/mL)	[EC50] (ug/mL)	[analysis] (ug/mL)	lag (hours)	elongation rate constant	endogenous in human systems?
1	heparin sodium salt	9041-08-1	573.40	17000 - 19000	-4	-6.98	-0.27	sugar	3 sulfates / 1 carboxyl	352 - 11	13.5	88	0.47	3.05	yes
2	chondroitin sulfate A ⌿	39455-18-0	475.38	6000 - 33000	-2	-4.21	-0.15	sugar	1 carboxyl / 1 sulfate	250 - 31.25	48.7	125	0.9	0.86	yes
3	hyaluronic acid (8-15 kD)	9067-32-7	402.31	8000 - 15000	-1	-2.49	-0.07	sugar	1 carboxyl	250 - 31.25	—	500.0	—	—	yes
4	hyaluronic acid (30-50 kD)	9067-32-7	402.31	30000 - 50000	-1	-2.49	-0.07	sugar	1 carboxyl	250 - 32.5	—	500.0	—	—	yes
5	hyaluronic acid (120-350 kD)	9067-32-7	402.31	120000 - 350000	-1	-2.49	-0.07	sugar	1 carboxyl	250 - 31.25	—	500.0	—	—	yes
6	hyaluronic acid (mixed mw)	9067-32-7	402.31	not availiable	-1	-2.49	-0.07	sugar	1 carboxyl	250 - 31.25	—	500.0	—	—	yes
7	fondaparinux	114870-03-0	1728.04	1728.04	-10	-5.79	-0.34	sugar	8 sulfates / 2 carboxyls	1000 - 31.25	72.5	500	3.17	0.18	no
8	nadroparin calcium Ψ	37270-89-6	591.45	—	-6	-5.07	-0.21	sugar	4 sulfates/ 2 carboxyl	1000 - 31.25	116.0	125	0.47	1.11	no
9	sodium alginate Ψ	9005-38-3	749.0	120000-190000	-2	-2.67	-0.12	sugar	2 carboxyls	1000 - 31.25	13.5	62.5	2.93	0.51	no
10	dermatan sulfate and oversulfated chondroitin sulfate Ψ	EPY0001321	475.38<br>696.52	not availiable	-2<br>-5	-4.21<br>-7.18	-0.15<br>-0.38	sugar	1 carboxyl / 1 sulfate<br>1 carboxyl / 4 sulfates	1000 - 61.25	49.3	250	0.2	1.77	yes
11	kappa carrageenan	11114-20-8	401.32	200000 - 800000	-1	-2.49	-0.08	sugar	1 sulfate	200 - 12.5	—	500.0	—	—	no
12	pectin	9000-69-5	353.3	50000 - 180000	-1	-2.83	-0.07	sugar	1 carboxyl	500 - 62.5	—	500.0	—	—	no
13	sodium pyrophosphate dibasic	7758-16-9	101.96	221.94	-1	-9.81	-0.31	phosphate	2 phosphates	4000 - 62.5	—	2000	—	—	yes
14	sodium pyrophosphate tetrabasic	7722-88-5	101.96	265.90	-1	-9.81	-0.31	phosphate	2 phosphates	4000 - 62.5	531.8	2000	2.85	0.25	yes
15	polyphosphate	10361-03-2	101.96	not availiable	-1	-9.81	-0.31	phosphate	1 phosphate	1000 - 125	146.3	250	0.67	1.04	yes
16	tripolyphosphate ┼	7758-29-4	101.96	367.85	-1	-9.81	-0.31	phosphate	3 phosphates	4000 - 62.5	—	2000	—	—	yes
17	Diadenosine pentaphosphate ┼	75522-97-3	101.96	916.37	-1	-9.81	-0.31	phosphate	5 phosphates	500 - 31.25	—	1000	—	—	yes
18	sodium hexametaphosphate Ψ	68915-31-1	101.96	611.77	-1	-9.81	-0.31	phosphate	6 phosphates	1000 - 15.6	65.1	125	2.07	0.22	no
19	sodium trimetaphosphate	7785-84-4	101.96	305.89	-1	-9.81	-0.31	phosphate	3 phosphates	4000 - 31.25	—	2000	—	—	no
20	Arachadonic acid	506-32-1	304.47	—	-1	-3.28	-0.12	lipid	1 carboxyl	30.5 - 1.9	—	31	0.75	0.37	yes
21	linoleic acid	60-33-3	280.45	—	-1	-3.57	-0.12	lipid	1 carboxyl	28.04 - 1.75	—	28	0.9	0.35	yes
22	phosphatidyl-l-serine	51446-62-9	792.07	—	-2	-2.53	-0.33	lipid	1 phospate / 1 carboxyl	18.5 - 1.16	—	79	1.1	0.29	yes
23	Gangloside disodium salt ⌿	37758-47-7	1470.80	—	-2	-1.36	-0.20	lipid	2 carboxyls	1000 - 31.25	—	100	—	—	yes
24	Gangloside diammonium salt ⌿	62010-37-1	1602.90	—	-2	-1.25	-0.20	lipid	2 carboxyls	1000 - 31.25	—	100	—	—	yes
25	poly-L-glutamic acid Ψ	26247-79-0	151.10	50000- 100000	-1	-6.62	-0.33	amino acid	1 carboxyl	500 - 15.6	26.0	62.5	0.57	2.48	yes
26	poly-L-glutamic acid (n = 20)	26247-79-0	151.10	3000.0	-1	-6.62	-0.33	amino acid	1 carboxyl	300 - 9.375	—	—	—	—	yes
27	poly-L-glutamic acid (n = 50)	26247-79-0	151.10	7500.0	-1	-6.62	-0.33	amino acid	1 carboxyl	300 - 9.375		75.0	0.4	0.55	yes
28	poly-L-glutamic acid (n = 100)	26247-79-0	151.10	15000.0	-1	-6.62	-0.33	amino acid	1 carboxyl	301 - 9.375		75.0	0.4	0.55	yes
29	poly-L-glutamic acid (n = 200)	26247-79-0	151.10	30000.0	-1	-6.62	-0.33	amino acid	1 carboxyl	302 - 9.375		75.0	0.2	0.52	yes
30	poly-L-lysine ┼	25104-18-1	151.10	150000 - 300000	1	6.62	0.33	amino acid	1 amine	500 - 31.25	—	250	—	—	yes
31	polyA (mRNA) ⌿	26763-19-9	267.24	not availiable	-1	-3.74	-0.11	nucleic acid	1 phosphate	1000 - 31.25	—	500	—	—	yes
32	Polyadenylic-polyuridylic acid (polyAU) ⌿	24936-38-7	671.40	not availiable	-2	-2.98	-0.06	nucleic acid	2 phosphates	500 - 62.5	—	500	—	—	yes
33	tRNA (yeast) ⌿	9014-25-9	—	25000.0	—	—	—	nucleic acid	—	500 - 62.5	—	500	2.1	0.49	yes
34	random nuc sequence	N8080127	—	—	—	—	—	nucleic acid	—	100 - 3.125	—	—	—	—	yes
35	polystyrene sulfonate Ψ	PSS4K	184.2	4800.0	-1	-5.43	-0.32	synthetic	1 sulfate	500 - 31.25	—	62.5	1.7	1.38	no
36	Fusidic acid sodium salt ⌿	751-94-0	538.7	—	-1	-1.86	-0.13	misc	1 carboxyl	1000 - 31.25	—	—	500	—	no
37	bilirubin	635-65-4	584.7	—	-2	-3.42	-0.10	misc	2 carboxyls	500 - 62.5	—	—	500	—	yes

(yellow)	active
┼	weak activator
(grey)	Inert
Ψ	hook effect
⌿	ThT reactivity

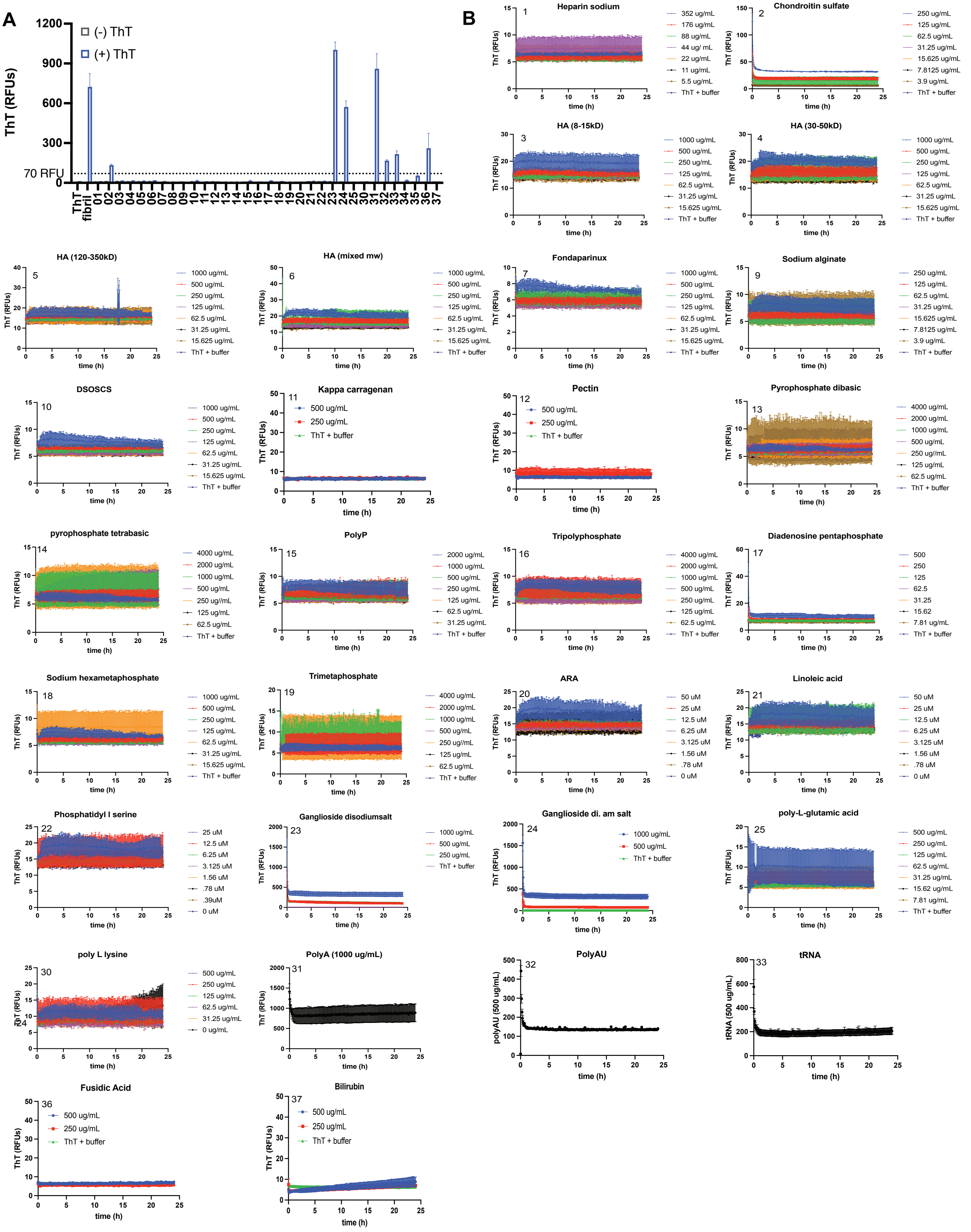

Supplementary Figure S1: Identification of anions that produce thioflavin T (ThT) artifacts inthe absence of tau fibrils. (A) Summary of the screen, in which anions were incubated with 10 μM ThT in the absence of fibrils. A subset, including chondroitin sulfate A (**2**), Ganglioside disodium salt (**23**), Ganglioside diammonium salt (**24**), polyA (mRNA) (**31**), Polyadenylic-polyuridylic acid (polyAU) (**32**), tRNA (**33**), and fusidic acid (**36**) produced ThT fluorescence in the absence of protein. **(B)** Raw data from the “no protein” controls. Results are the average of technical triplicates and error is SEM.

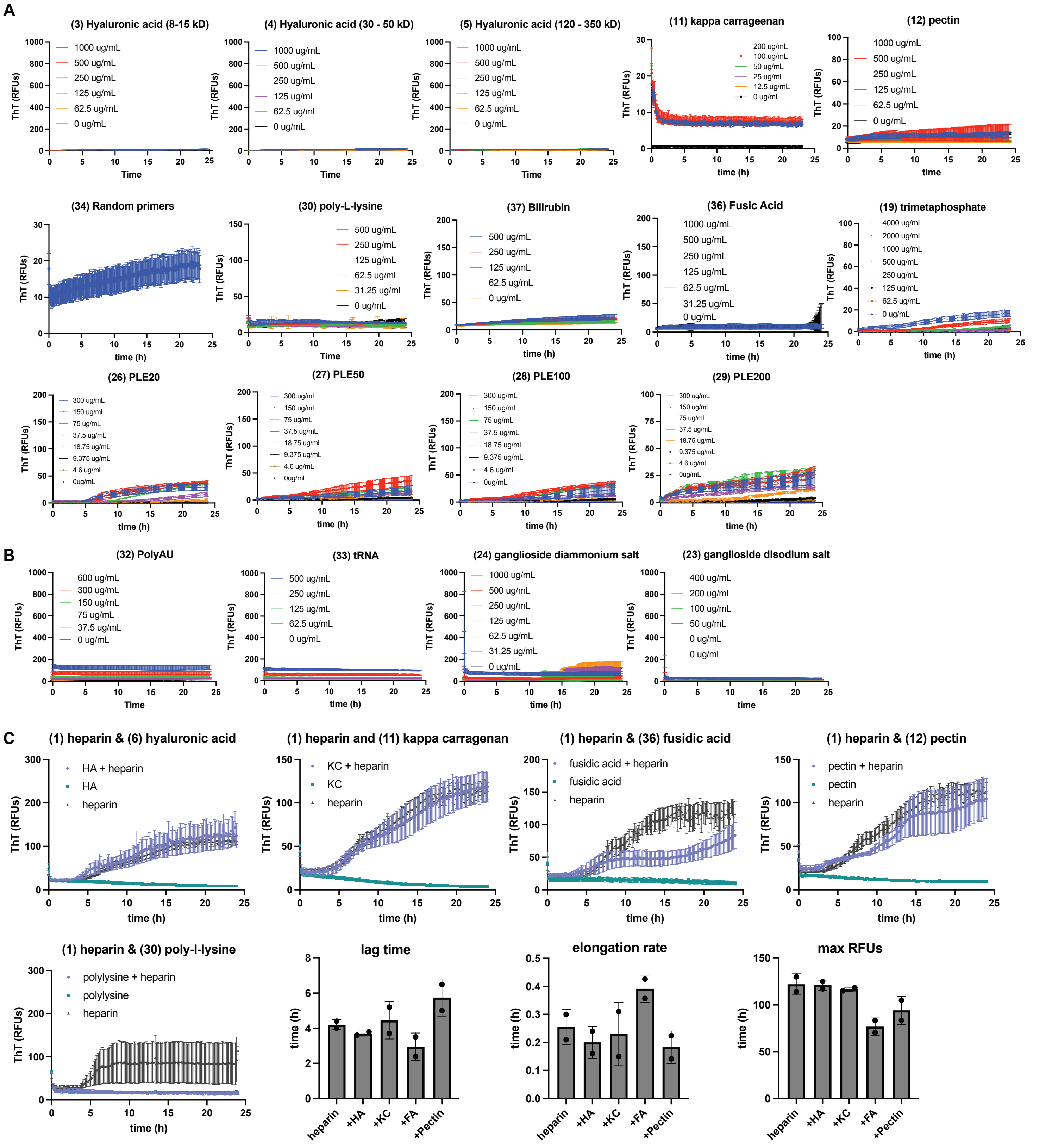

Supplementary Fig. S2. Raw curves supporting the activity of anions in the ThT assays. (A) Results from ThT assays for the subset of anions that are unable to promote tau aggregation (termed “inert”). (B) Results from ThT assays for the anions that have high background. For these anions, we rely more heavily on the sedimentation and TEM experiments. (C) A subset of the “inert” anions (36, fusidic acid; 12, pectin) are able to partially inhibit the activity of heparin when added simultaneously, while others (6, hyaluronic acid; 11 kappa-carragenan) are not. All results are the average of technical triplicates and error is SEM. Results are the average of two independent experiments performed in triplicate and error is SEM.

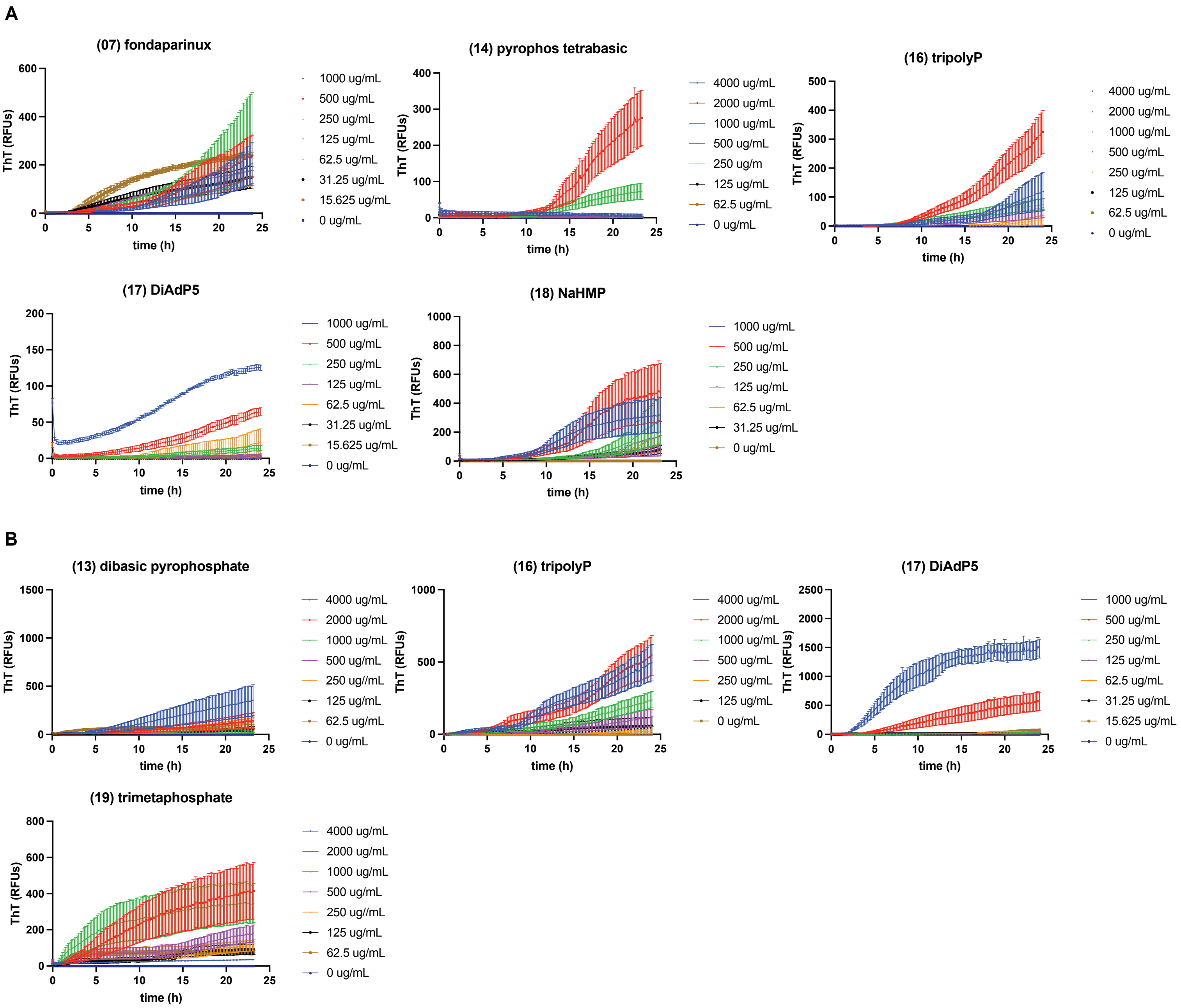

Supplementary Fig. S3 A subset of anions weakly induce tau aggregation. Raw ThT plots for the subset of anions that were designated as “weakly active” for (A) WT tau and (B) P301S tau. Results are the average of at least three independent experiments performed in triplicate and error is SEM.

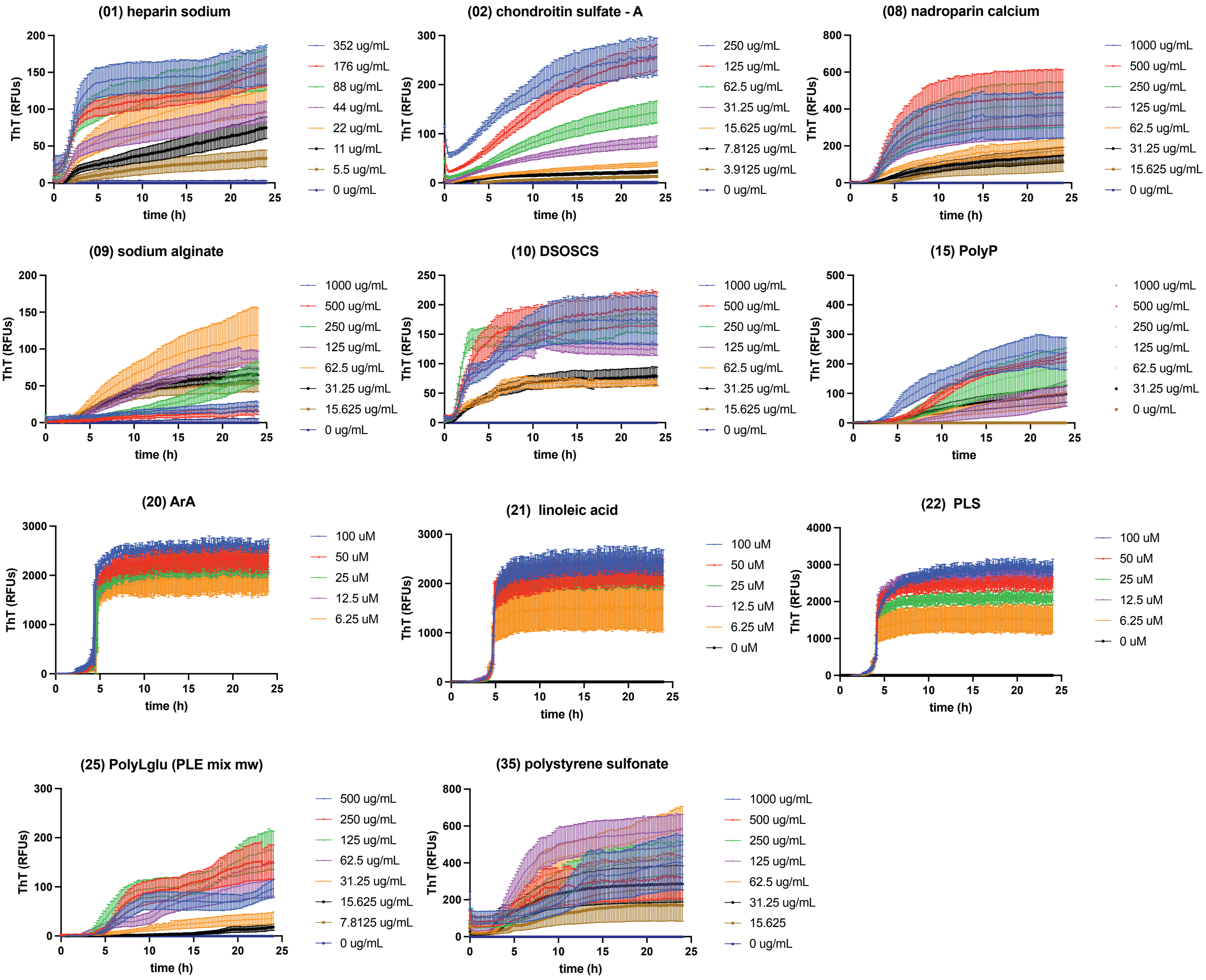

Supplementary Fig. S4. Raw ThT results for the anions determined to be "active" against WT tau. For these anions, we performed full kinetic and dose range screening. Results are the average of at least three independent experiments performed in triplicate and the error bars represent SEM (n=9).

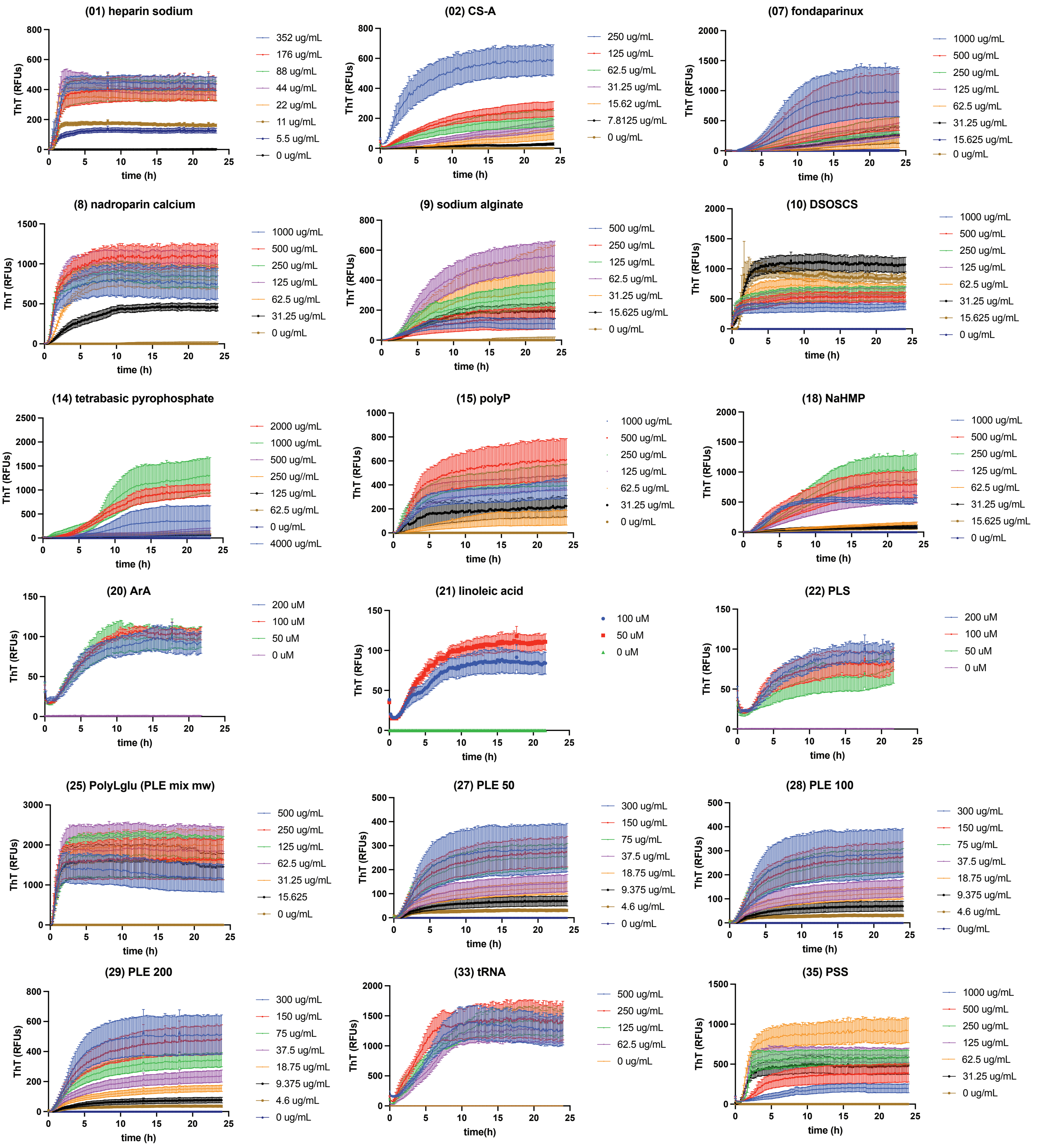

Supplementary Fig. S5. Raw ThT results for the anions determined to be "active" against P301S tau. For these anions, we performed full kinetic and dose range screening. Results are the average of at least three independent experiments performed in triplicate and the error bars represent SEM (n=9).

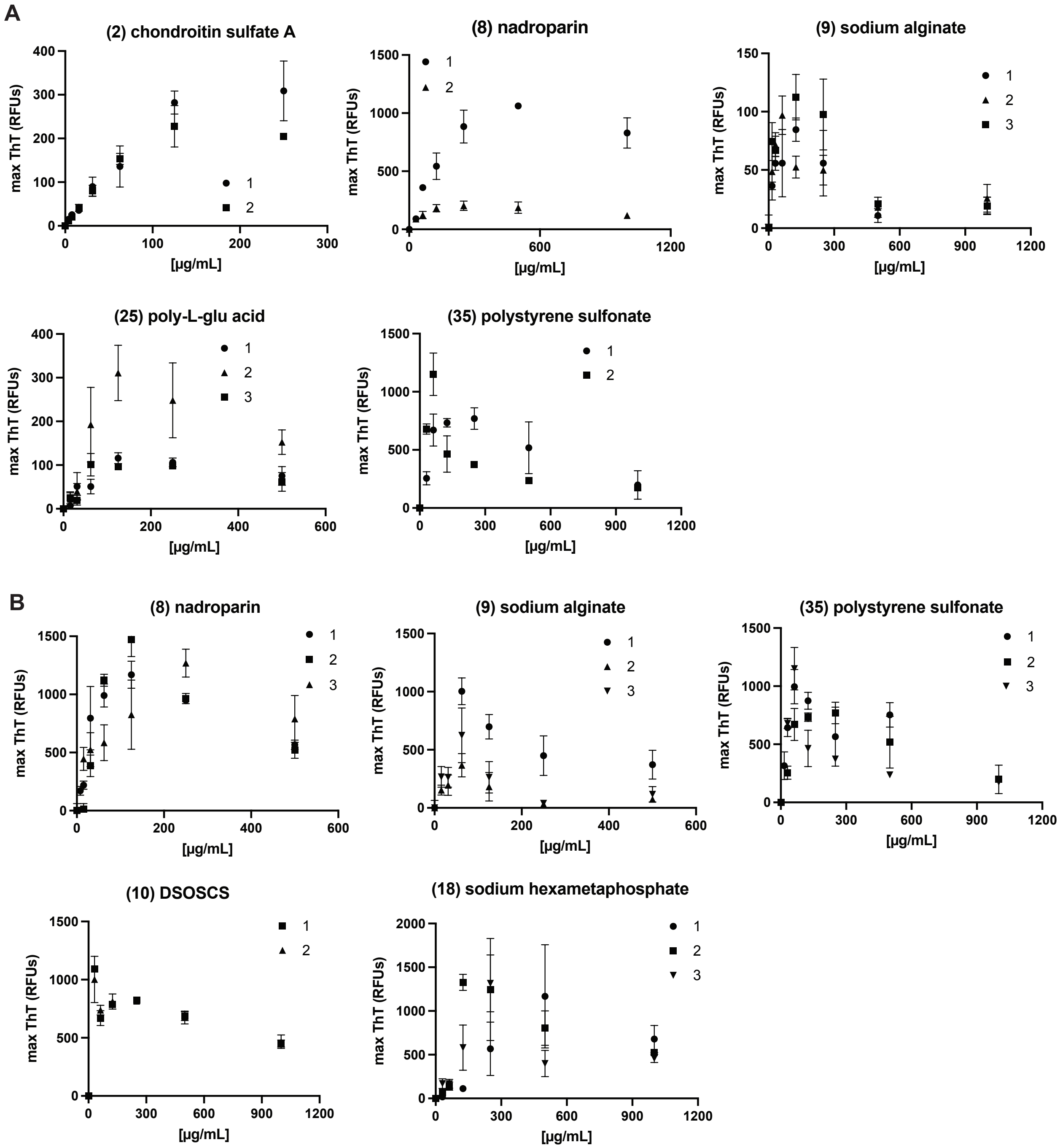

Supplementary Fig. S6 Several anions accelerate aggregation at low concentrations, but then inhibit it at higher concentrations (producing a "hook effect"). Raw ThT results are shown for (A) WT Tau and (B) P301S tau. Each curve represents are the average of three experiments performed in technical triplicate and the error bars represent SEM (n=3). The two independent, biological replicates are shown (1, 2, etc.). As stated in the text, these inducers were used at EC50 values that were selected after excluding the higher concentrations.

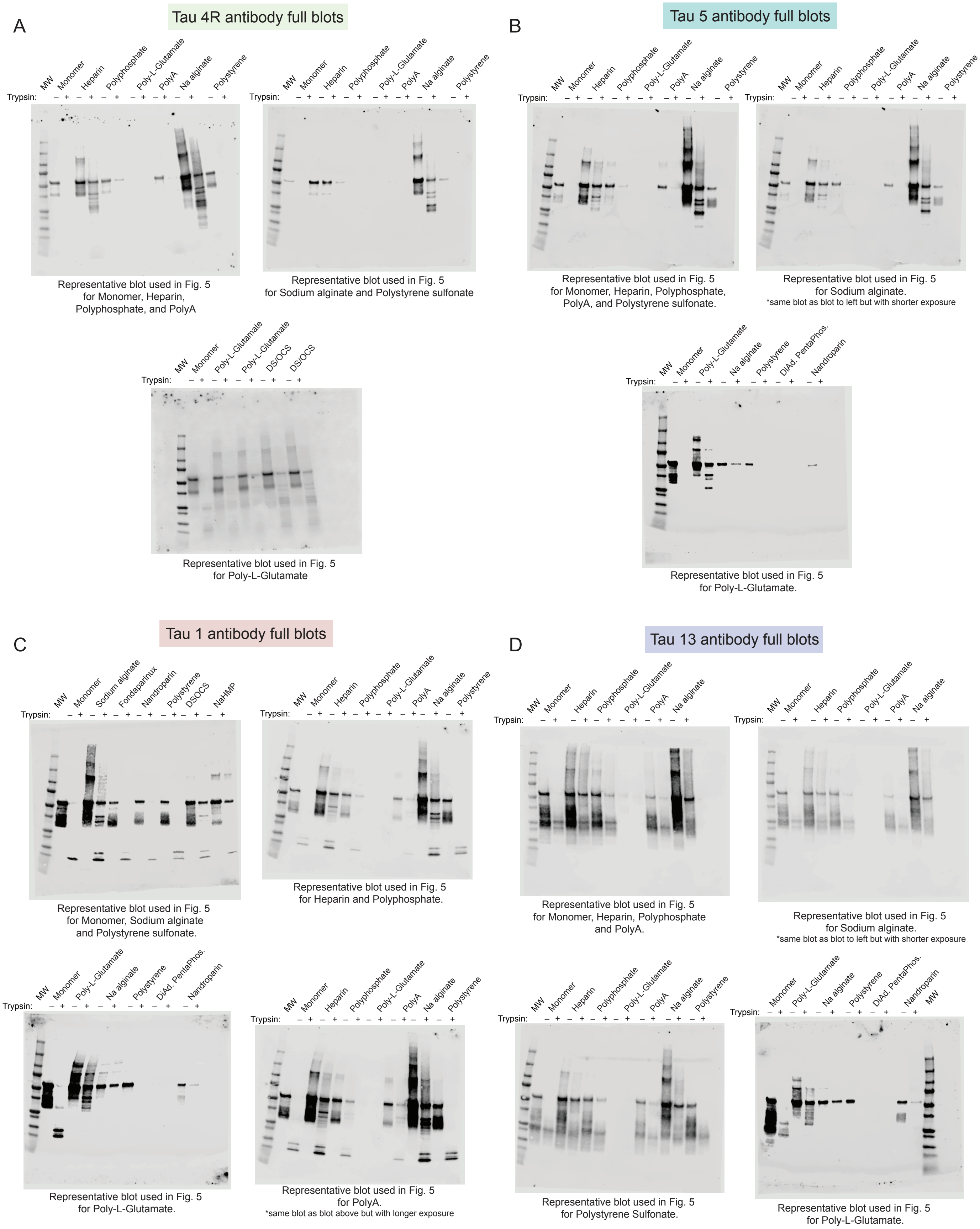

Supplementary Fig. S7 Full Western blots for the images shown in the partial proteolysis studies (see Figure 4). The full, uncropped Western blots for the partial proteolysis studies, using antibodies (a) Tau 4R (b) Tau 5 (c) Tau 1 and (d) Tau 13. See text for details.

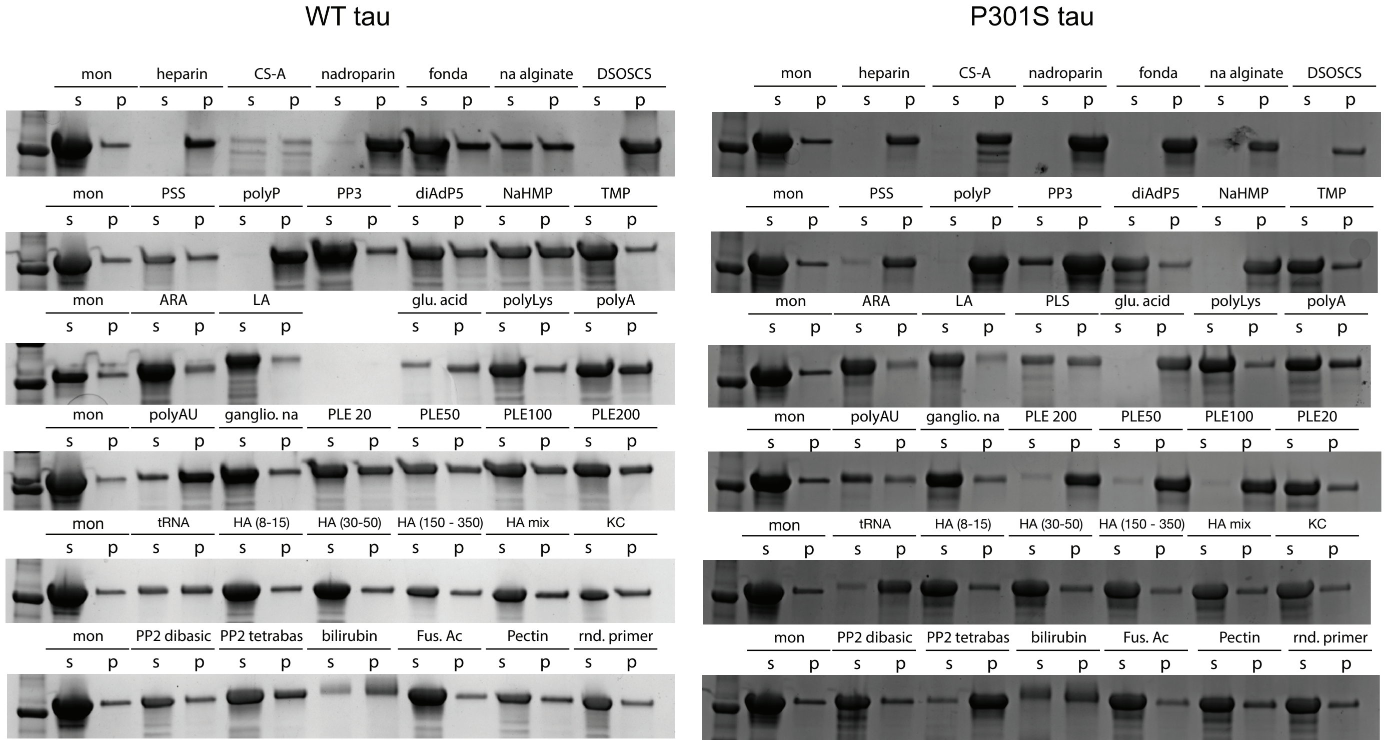

Supplementary Fig. S8. Sedimentation assays reveal the extent of tau fibrilization. After incubation of tau with anions, centrifugation was used to separate the insoluble pellet (P) and soluble (S) components, followed by analysis by SDS-PAGE. The concentration of anion used in these studies is the “analysis concentration”, as denoted in SI Tables 1-2.

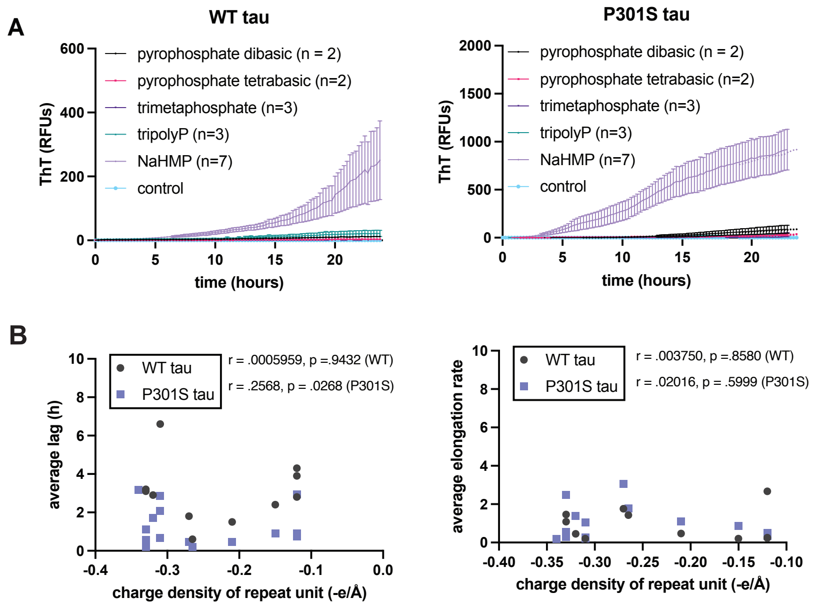

Supplementary Fig. S9. Polyanion valency is an important parameter in dictating tau fibril formation. (A) 2N solutions of polyphosphates including pyrophosphate (13-14), tripolyphosphate (16), sodium hexametaphosphate (18) or sodium trimetaphosphate (19) were used to induce aggregation of WT (left) or P301S tau (right) . The relative valency is indicted (n=x). These results further support the conclusions in Figure 3. (B) Correlation analysis of the effect of charge density (as determined by approximate Angstrom distance of the repeat unit (-e/Å) on lag time (left) and elongation rate (right) using a Pearson t-test, demonstrating that these parameters do not correlate for either WT or P301S tau. These results support the conclusions in Figure 3.

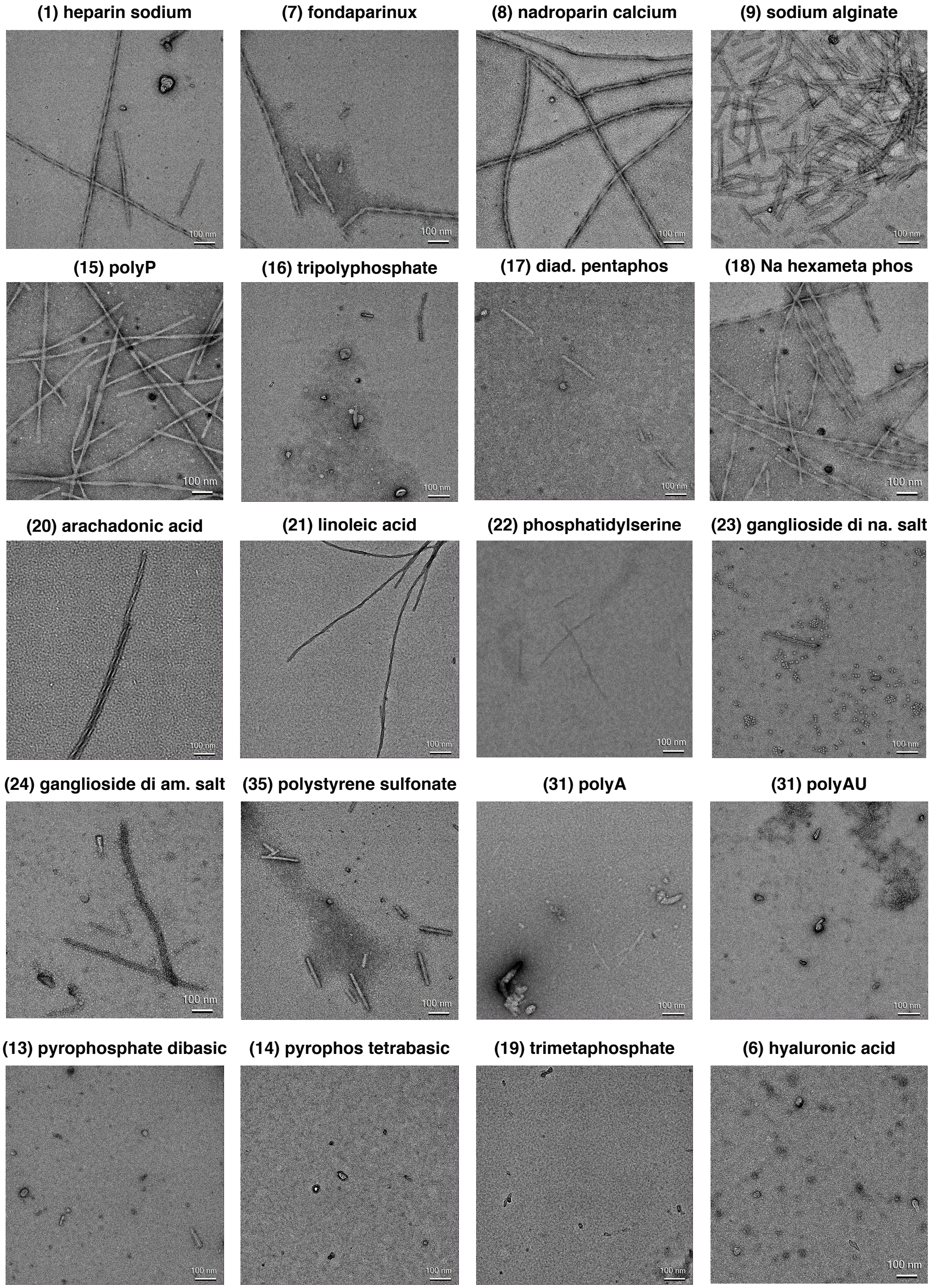

Supplementary Fig. S10. Raw TEM images, supporting and extending the results from the ThT and sediminatation assays. Results are representative of collected images. Results are shown for WT tau. Inactives and weakly actives are grouped at the bottom. Scale bar is 100 nm.

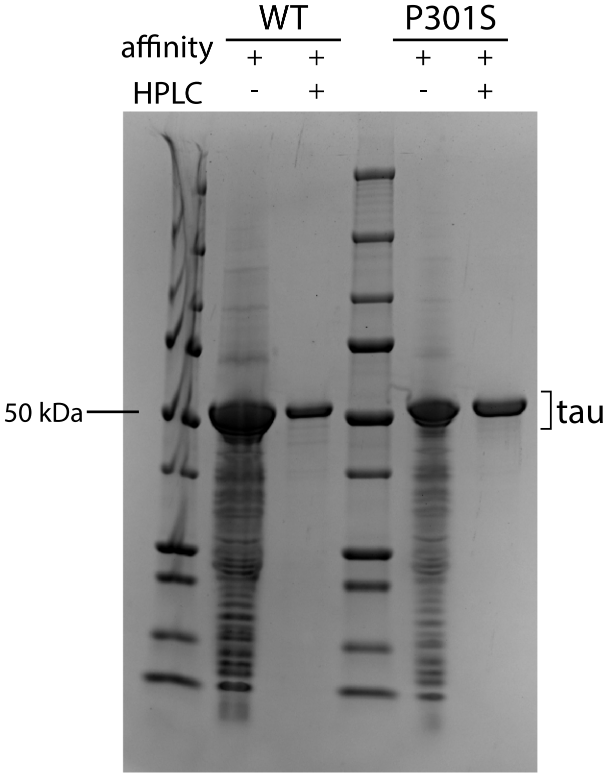

Supplementary Figure S11. Purity of expressed tau proteins, as judged by Coomassie gels. The samples are shown after the His-tag affinity step and after the final HPLC purification, for both the WT 0N4R tau and P301S 0N4R tau. The samples were 95% pure, as measured by ImageJ.
